# Supplementary material for: Genotypic, functional, and phenotypic characterization in CTNNB1 neurodevelopmental syndrome
Source: HGG Adv. 2025 Jul 18;6(4):100483. doi: 10.1016/j.xhgg.2025.100483 (PMC12335994; doi:10.1016/j.xhgg.2025.100483)
Supplement: Document S1. Figures S1–S8 and Tables S4–S17 [file mmc1.pdf]

HGGA, Volume 6

Supplemental information

Genotypic, functional, and phenotypic characterization in CTNNB1 neurodevelopmental syndrome

Nina Žakelj, David Gosar, Špela Miroševič, Stephan J. Sanders, Alicia Ljungdahl, Sayeh Kohani, Shouhe Huang, Lok I Leong, Ying An, Miou-Jing Teo, Fiona Moultrie, Roman Jerala, Duško Lainšček, Vida Forstnerič, Petra Sušjan, Leszek Lisowski, Andrea Pérez-Iturralde, Jasna Oražem Mrak, Ho Yin Edwin Chan, and Damjan Osredkar

Supplementary Text

VSS: "The four levels include: (1) speech is not affected by motor disorder; (2) speech is imprecise but usually understandable to unfamiliar listeners; (3) speech is unclear and not usually understandable to unfamiliar listeners out of context; (4) no comprehensible speech. "

FCSS: "The five levels include: (1) effective communicator in most situations; (2) effective communicator in most situations, but does need some help; (3) an effective communicator in most situations and can communicate small ranges of messages and topics to most familiar people; (4) assistance is required in most situations, especially with unfamiliar people and environments; (5) communicates unintentionally using movements and behaviour."

GMFCS: "The five levels include: (1) Children walk at home, school, outdoors and in the community. They can climb stairs without the use of a railing. Children perform gross motor skills such as running and jumping, but speed, balance and coordination are limited; (2) Children walk in most settings and climb stairs holding onto a railing. They may experience difficulty walking long distances and balancing on uneven terrain, inclines, in crowded areas or confined spaces. Children may walk with physical assistance, a handheld mobility device or used wheeled mobility over long distances. Children have only minimal ability to perform gross motor skills such as running and jumping; (3) Children walk using a hand-held mobility device in most indoor settings. They may climb stairs holding onto a railing with supervision or assistance. Children use wheeled mobility when travelling long distances and may self-propel for shorter distances; (4) Children use methods of mobility that require physical assistance or powered mobility in most settings. They may walk for short distances at home with physical assistance or use powered mobility or a body support walker when positioned. At school, outdoors and in the community children are transported in a manual wheelchair or use powered mobility; (5) Children are transported in a manual wheelchair in all settings. Children are limited in their ability to maintain antigravity head and trunk postures and control leg and arm movements."

VFCS: (1) Uses visual function easily and successfully in vision-related activities; (2) Uses visual function successfully but needs self-initiated compensatory strategies; (3) Uses visual function but needs some adaptations; (4) Uses visual function in very adapted environments but performs just part of vision-related activities; (5) Does not use visual function even in very adapted environments.

EADCS: (1) Eats and drinks safely and efficiently; (2) Eats and drinks safely but with some limitations to efficiency; (3) Eats and drinks with some limitations to safety. There maybe limitations to efficiency; (4) Eats and drinks with significant limitations to safety; (5) Unable to eat or drink safely - tube feeding may be considered to provide nutrition.

Supplementary Figures

Figure S1. Alternative splicing transcripts for the *CTNNB1*. Genomic coordinates GRCh38/hg38 for a 49k nucleotide region of chromosome 3 are shown at the top. There is a ~13k gap to help highlight regions of interest. Four features of note are named in red with light red bars showing the genomic region across the figure. Nucleotide-resolution of the median read counts of RNA-seq gene expression data from 176 *postmortem* human frontal cortices (PMID: 32268104) are shown organized by developmental stage; data are auto-scaled. The protein-coding (CDS) exon number is show for comparability to Fig. 2. All GENCODEv46 transcripts for the *CTNNB1* gene are represented, including the MANE Select transcript ENST00000349496.11, NM_001904.4 at the top in light blue. Protein-coding transcripts are in dark blue, while non-coding transcripts are shown in grey. Epigenetic data from ENCODE, specifically the H3K27ac marker of actively expressed regions across cell types, is represented under the gene transcripts. Species conservation across 100 vertebrates is represented at the bottom, including PhyloP scores (top) and homology across species (bottom). We note that the TSS region is shown as absent in *Rhesus*, however, BLAT of the human TSS DNA sequence correctly identifies the corresponding TSS region in *Rhesus*, suggesting a cross-species genome alignment error in this representation. Image was generated from UCSC genome browser (PMID: 36420891).

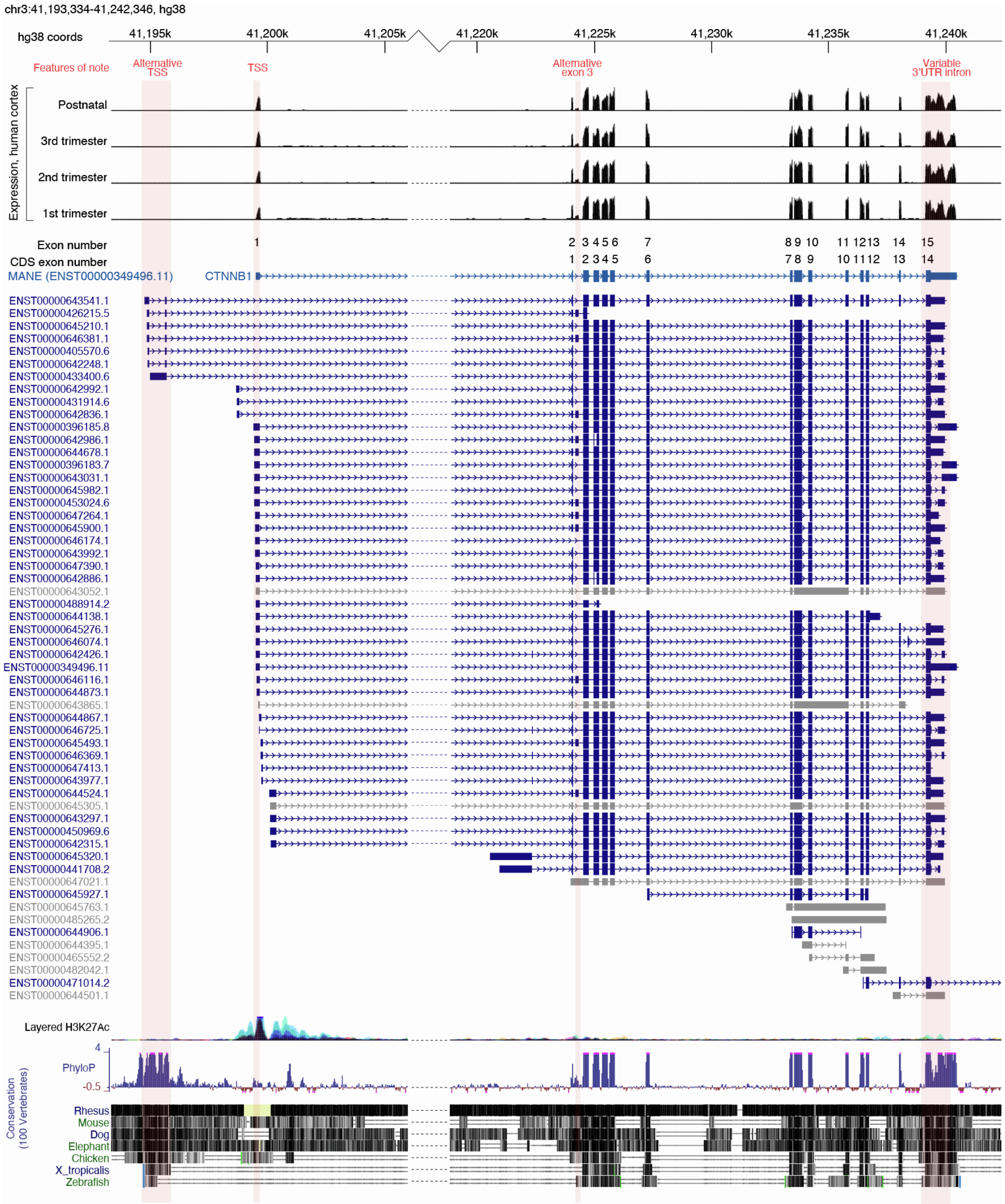
chr3:41,193,334-41,242,346, hg38
hg38 coords
41,195k
41,200k
41,205k
41,220k
41,225k
41,230k
41,235k
41,240k
Features of note
Alternative TSS
TSS
Alternative exon 3
Variable 3'UTR intron
Expression, human cortex
Postnatal
3rd trimester
2nd trimester
1st trimester
Exon number
CDS exon number
MANE (ENST00000349496.11)
CTNNB1
ENST00000643541.1
ENST00000426215.5
ENST00000645210.1
ENST00000646381.1
ENST00000405570.6
ENST00000642248.1
ENST00000433400.6
ENST00000642992.1
ENST00000431914.6
ENST00000642836.1
ENST00000396185.8
ENST00000642986.1
ENST00000644678.1
ENST00000396183.7
ENST00000643031.1
ENST00000645982.1
ENST00000453024.6
ENST00000647264.1
ENST00000645900.1
ENST00000646174.1
ENST00000643992.1
ENST00000647390.1
ENST00000642886.1
ENST00000643052.1
ENST00000488914.2
ENST00000644138.1
ENST00000645276.1
ENST00000646074.1
ENST00000642426.1
ENST00000349496.11
ENST00000646116.1
ENST00000644873.1
ENST00000643865.1
ENST00000644867.1
ENST00000646725.1
ENST00000645493.1
ENST00000646369.1
ENST00000647413.1
ENST00000643977.1
ENST00000644524.1
ENST00000645305.1
ENST00000643297.1
ENST00000450969.6
ENST00000642315.1
ENST00000645320.1
ENST00000441708.2
ENST00000647021.1
ENST00000645927.1
ENST00000645763.1
ENST00000485265.2
ENST00000644906.1
ENST00000644395.1
ENST00000465552.2
ENST00000482042.1
ENST00000471014.2
ENST00000644501.1
Layered H3K27Ac
4
PhyloP
-0.5
Conservation (100 Vertebrates)
Rhesus
Mouse
Dog
Elephant
Chicken
X_tropicalis
Zebrafish

Figure S2. Developmental patterns of exon expression for the *CTNNB1*. A) The expression of 128 exon subdivisions, as defined by GENCODE transcripts and DEX-seq (Table SX), are represented across development. The lines represent the Lowess smoothed log-scaled counts per million (CPM) values across development from 176 *postmortem* human frontal cortices (PMID: 32268104). **B)** The correlation between the ratio of CPM expression between pairs of protein-coding exon subdivisions (log-scaled) and developmental stage (post-conceptual days, log-scaled) for *CTNNB1* was assessed with a linear model. The R^2 value of each exon pair is shown as a heat map with 'hot' colors representing exon pairs with high R^2 values for which variation in the ratio is correlated with developmental age, i.e., pairs of exons that show substantially different expression across development (PMID: 34425903).

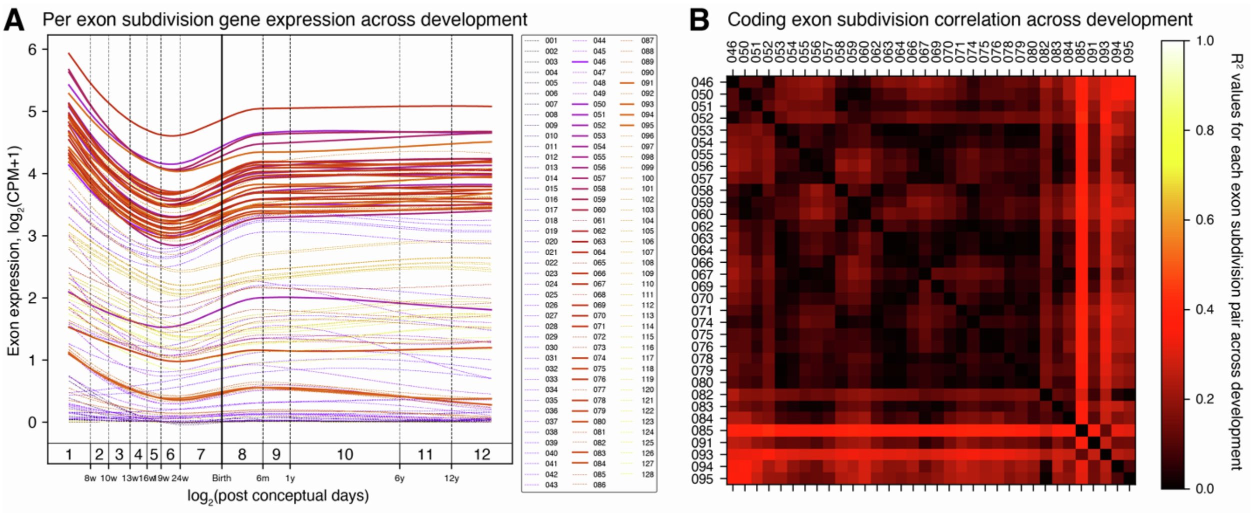

Figure S3. Number of participants in clinical trial per country and the world-map of countries with involved participants.

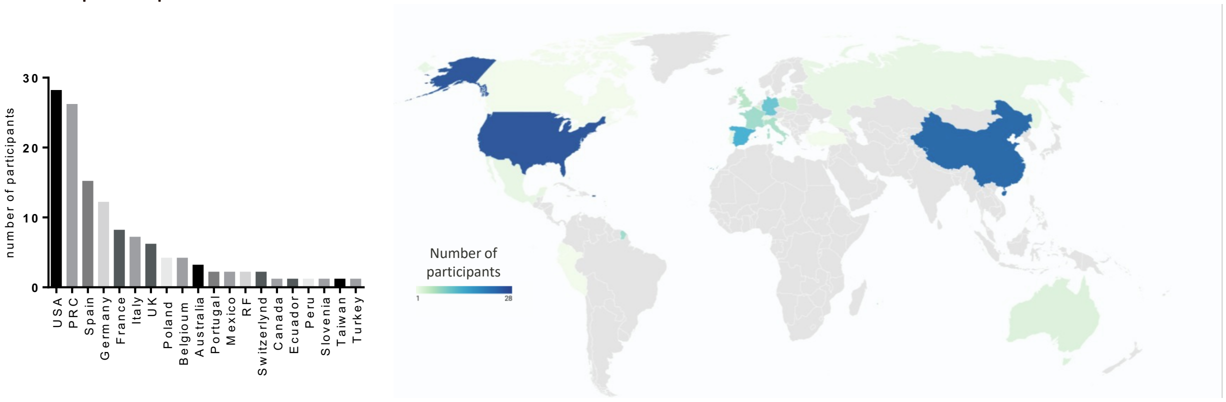

Figure S4. β-catenin protein expression in various CTNNB1 mutant constructs. WT – wildtype.

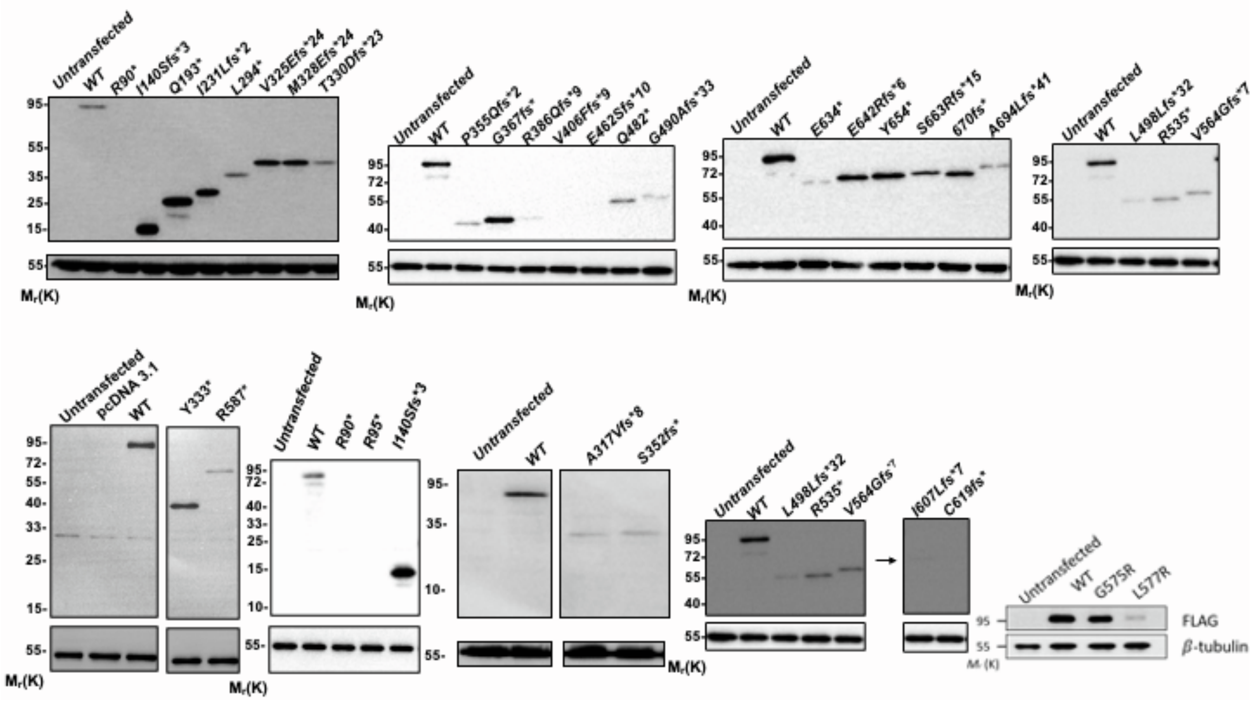

Figure S5. Thermal stability of β-catenin mutants determined by cellular thermal shift assay (CETSA). A) Cellular proteins extracted from SK-N-MC cells expressing *CTNNB1^{WT}, CTNNB1^{G575R}, or CTNNB1^{L577R}* were exposed to temperatures ranging from 40°C to 68°C and the soluble fractions were collected for western blot analysis. **B)** The relative band intensity of FLAG signal was quantified and fitted using the Boltzmann sigmoidal equation. Results are from three independent experiments. Error bars denote standard error of the mean (SEM). *Tm, melting temperature; ICE, ice control; RT, room temperature.*

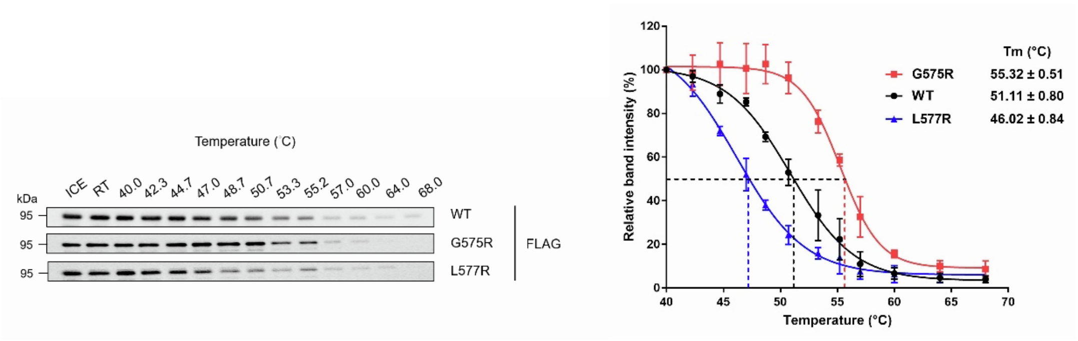

Figure S6. Prenatal and perinatal characteristics. Percentage of (A) prenatal and (B) perinatal risk factors in CTNNB1 syndrome participants with 95% confidence intervals.

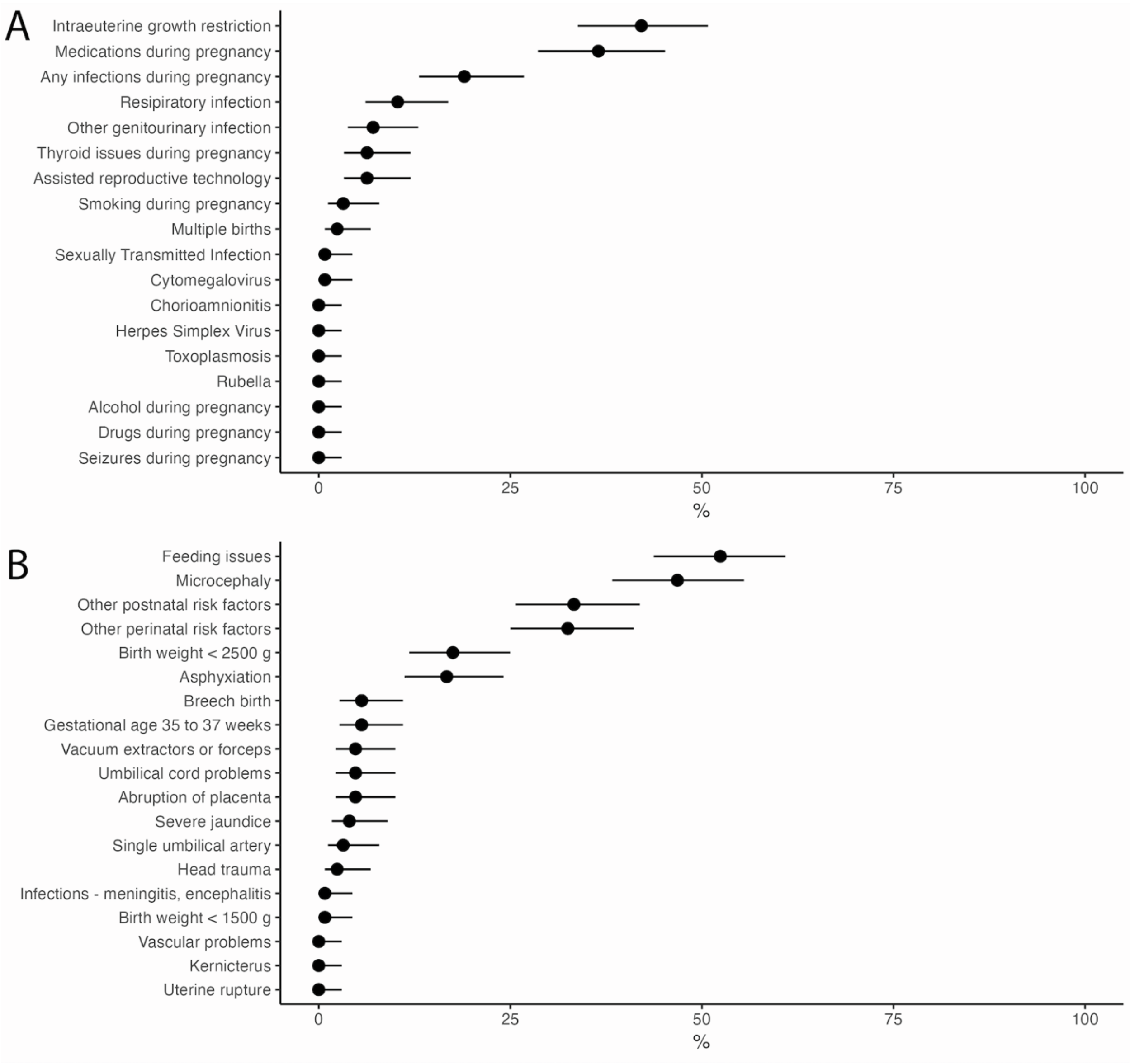

Figure S7. Ophthalmologic signs/symptoms in CTNNB1 syndrome patients as reported by parents with 95% confidence intervals.

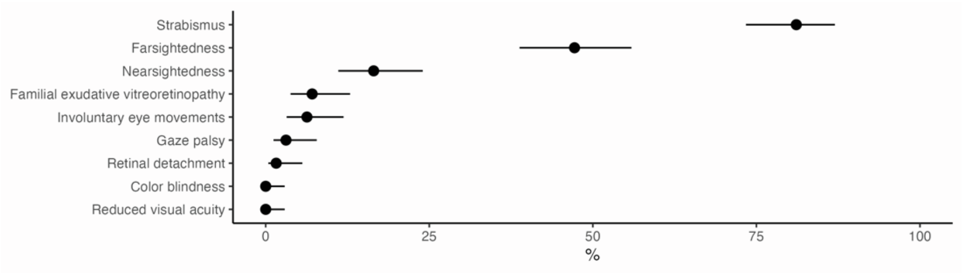

Figure S8. ABAS-3 scores for adaptive skills subdomains (A) and adaptive skills domains (B) for CTNNB1 syndrome patients (N = 114).

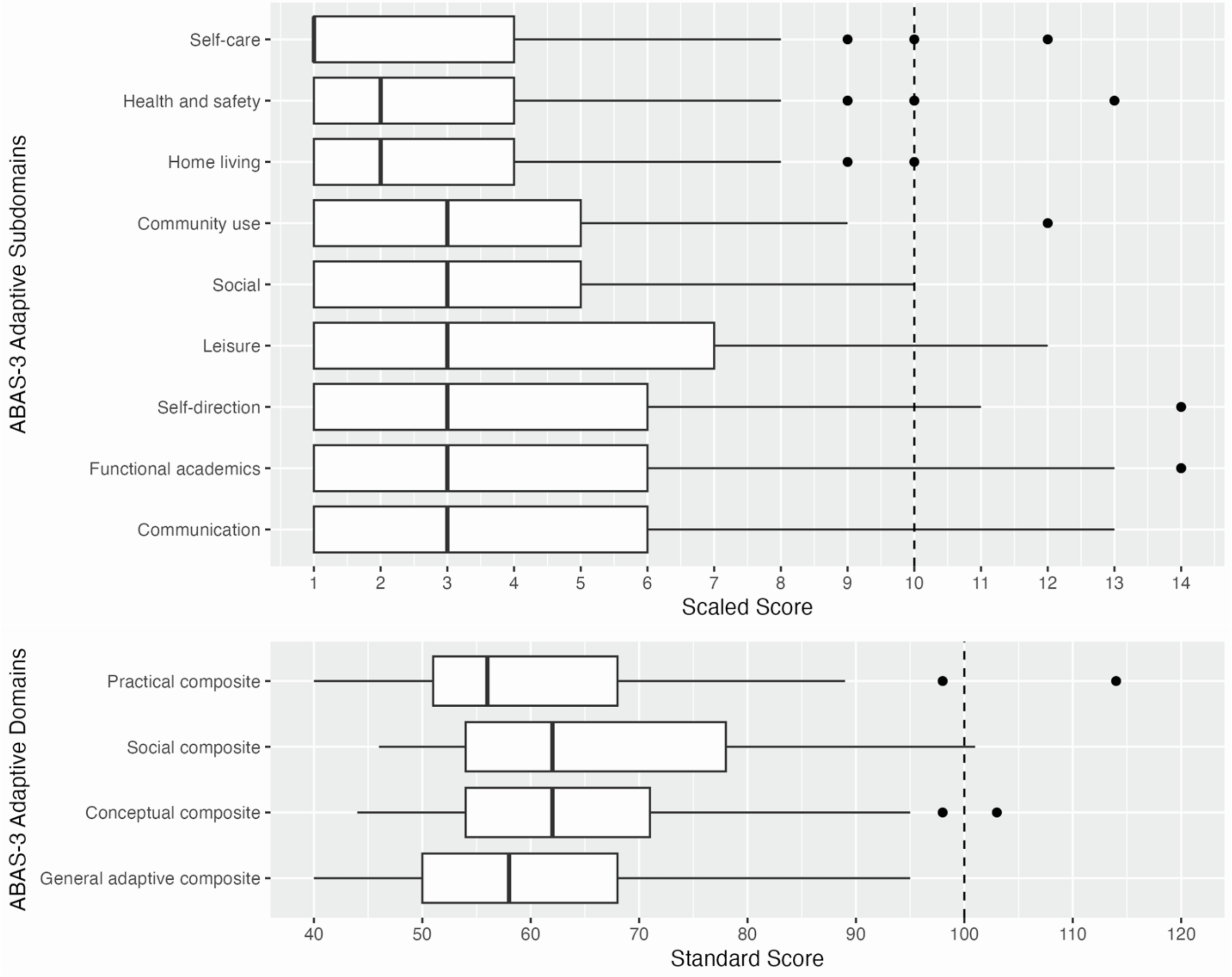

Supplementary Tables

Table S1. All SNVs (separate file)
Supplementary Table 1.xlsx

Table S2. Variant Mapper and Clinical Data (separate file)
Supplementary Table 2.xlsx

Table S3. Exon subdivisions (separate file)
Supplementary Table 3.xlsx

Table S4. Estimated protein size of overexpressed mutant β-catenin proteins.

Mutant protein (overexpressed)	Observed protein size (kDa)
R90*	Not detected
R95*	Not detected
I140Sfs*3	~15
Q193*	~25
I231Lfs*2	~27
L294*	~35
V325Efs*24	~38
M328Efs*24	~38
T330Dfs*23	~38
Y333*	~38
P355Qfs*2	~40
G367fs*	~42
R386Qfs*9	~42
V406Ffs*9	Not detected
E462Sfs*10	Not detected
Q482*,	~52
G490Afs*33	~54
L498Lfs*32,	~54
R535*,	~54
V564Gfs*7	~56
R587*	~64
I607Lfs*7	~67
C619fs*	Not detected
E634*	~69
E642Rfs*6	~70
Y654*	~70
S663Rfs*15	~71
A694Lfs*41	~72
Y670fs*	~70

Table S5. Standardized scores on the ABAS-3 adaptive skill domains and the General Adaptive Composite (GAC) of patients with different types of *CTNNB1* variants. *Me* – median of posterior distribution*; HDI* low – lower bound of the 89% highest density interval for the posterior distribution;*HDI* high – upper bound of the 95% highest density interval for the posterior distribution; *p*d – probability of direction (likelihood of an effects existence); ROPE – proportion outside of Region of Practical Equivalence for neurotypical individuals (ROPE).

	Me	*HDI* low	*HDI* high	*pd*	*ROPE*
**General Adaptive Composite**					
Missense variant	68.6	46.8	88.3	<.001	<.001
Nonsense variant	52.4	36.8	62.6	<.001	.001
Frameshift variant	47.6	31.5	58.6	<.001	.001
Whole gene deletion	63.8	39.2	86.8	.400	.004
Splice variant	55.4	35.7	72.0	.100	.003
**Conceptual Domain**					
Missense variant	84.6	69.0	97.9	<.001	<.001
Nonsense variant	63.4	59.9	67.0	<.001	.001
Frameshift variant	64.1	60.7	67.4	<.001	<.001
Whole gene deletion	60.5	41.0	73.3	<.001	.001
Splice variant	62.2	50.4	70.5	<.001	<.001
**Social Domain**					
Missense variant	83.0	67.0	95.7	<.001	<.001
Nonsense variant	66.8	63.4	70.3	<.001	.001
Frameshift variant	64.6	60.4	68.2	<.001	.001
Whole gene deletion	67.4	51.1	79.2	.100	.002
Splice variant	63.9	52.3	73.6	<.001	.001
**Practical Domain**					
Missense variant	63.5	39.9	85.3	<.001	<.001
Nonsense variant	50.8	34.1	62.9	.100	.004
Frameshift variant	40.8	23.5	56.0	<.001	.001
Whole gene deletion	73.7	45.9	99.4	.300	.003
Splice variant	57.9	36.8	76.0	.300	.005

Table S6. Censored regression results for predicting age at indepedent walking based on functional status of variant (LoF dominant negative vs. LoF not dominant negative).

	LoF DN			LoF nDN				
		95% HDI			95% HDI			
	Est.	lw. bound	up. bound	Est.	lw. bound	up. bound	*BF*	*pd*
Age at independent walking (in months)	36.0	23.7	51.7	33.7	27.7	40.4	1.65	0.622

Note: LoF DN - LoF dominant negative, LoF nDN - LoF not dominant negative

Table S7. Regression results for predicting adaptive skills based on functional status of variant (LoF dominant negative vs. LoF not dominant negative).

	LoF DN			LoF nDN				
		95% HDI			95% HDI			
	Est.	lw. bound	up. bound	Est.	lw. bound	up. bound	*BF*	*pd*
ABAS-3 Scales								
GAC	54.1	38.1	66.8	53.7	40.5	63.0	0.81	0.449
Conceptual Composite	61.2	49.2	70.7	62.8	54.1	68.9	1.63	0.619
Social Composite	64.5	51.2	74.6	63.1	50.3	69.8	0.53	0.347
Practical Composite	49.2	24.1	66.0	53.4	36.3	64.3	2.10	0.678

Note: LoF DN - LoF dominant negative, LoF nDN - LoF not dominant negative

Table S8. Regression results for predicting symptoms of psychopathology based on functional status of variant (LoF dominant negative vs. LoF not dominant negative).

	LoF DN			LoF nDN				
		95% HDI			95% HDI			
	Est.	lw. bound	up. bound	Est.	lw. bound	up. bound	*BF*	*pd*
ASEBA Scales								
Anxious/Depressed	57.9	53.5	61.9	60.1	57.8	62.7	6.83	0.872
Withdrawn Behavior	61.8	55.9	67.4	65.7	62.6	68.8	8.10	0.890
Somatic Complaints	58.0	54.4	61.5	58.2	56.1	60.5	1.19	0.543
Thought Problems	75.3	64.6	85.2	72.9	67.8	78.2	0.52	0.342
Autism Spectrum	62.2	56.6	68.1	66.7	63.5	69.8	9.46	0.904
Attention Problems	66.2	60.8	71.7	69.5	66.6	72.5	6.25	0.862
Aggressive Behavior	60.4	55.7	65.1	62.0	59.2	65.1	3.05	0.753

Note: LoF DN - LoF dominant negative, LoF nDN - LoF not dominant negative

Table S9. Ordinal regression results for predicting the functional assessment scale scores of patientswith a LoF dominant negative variant compared to patients with a dominant negative variant.

		95% HDI			
	Est.	lw. bound	up. bound	*BF*	*pd*
Functional Scales					
VSS	-0.66	-2.55	1.36	3.1	0.754
FCCS	-1.01	-2.76	0.77	6.9	0.873
GMFCS	-0.44	-1.65	0.83	3.1	0.756
VFCS	-0.2	-1.54	1.12	1.6	0.621
EDACS	-0.15	-1.8	1.49	1.3	0.571

Note: GMFCS – Gross Motor Function Classification System; EADCS – Eating and Drinking Ability Classification System; FCCS – Functional Communication Classification System; VFCS – Visual Function Classification System; VSS – Viking speech scale

Table S10. Censored regression results for the age at independent walking by patients with a LoF dominant negative variant compared to patients with a dominant negative, LoF or LoF presumed variant.

	LoF DN			LoF nDN+				
		95% HDI			95% HDI			
	Est.	lw. bound	up. bound	Est.	lw. bound	up. bound	*BF*	*pd*
Age at independent walking (in months)	36.0	23.7	51.7	39.3	35.1	43.6	0.48	0.323

Note: LoF DN - LoF dominant negative, LoF nDN+ - LoF not dominant negative, LoF, LoF presumed.

Table S11. Regression results for predicting the ABAS-3 adaptive skills of patients with a LoF dominant negative variant compared to patients with a dominant negative, LoF or LoF presumed variant.

	LoF DN			LoF nDN+				
		95% HDI			95% HDI			
	Est.	lw. bound	up. bound	Est.	lw. bound	up. bound	*BF*	*pd*
ABAS-3 Scales								
GAC	51.3	31.8	66.3	53.5	39.5	60.9	1.35	0.575
Conceptual Composite	61.7	51.0	68.9	63.8	61.3	66.5	2.93	0.746
Social Composite	65.6	58.0	72.1	65.5	63.0	68.2	0.96	0.489
Practical Composite	50	26.3	65.8	54.8	43.2	61.6	2.21	0.689

Note: LoF DN - LoF dominant negative, LoF nDN+ - LoF not dominant negative, LoF, LoF presumed.

Table S12. Regression results for predicting symptoms of psychopathology in patients with a LoF dominant negative variant compared to patients with a dominant negative, LoF or LoF presumed variant.

	LoF DN			LoF nDN+				
		95% HDI			95% HDI			
	Est.	lw. bound	up. bound	Est.	lw. bound	up. bound	*BF*	*pd*
ASEBA Scales								
Anxious/Depressed	58.6	54.3	61.8	60.5	59.1	62.1	8.72	0.897
Withdrawn Behavior	62.5	56.7	68.3	65.6	63.6	67.5	5.63	0.849
Somatic Complaints	58.3	55.0	61.0	58.8	57.5	60.3	1.77	0.639
Thought Problems	73.9	63.8	84.4	73.2	70.3	76.0	0.82	0.451
Autism Spectrum	62.1	55.0	68.8	69.1	66.7	71.5	26.93	0.964
Attention Problems	66.6	60.6	72.0	69.4	67.5	71.3	4.65	0.823
Aggressive Behavior	61.0	56.2	65.0	63.1	61.3	65.1	6.43	0.865

Note: LoF DN - LoF dominant negative, LoF nDN+ - LoF not dominant negative, LoF, LoF presumed.

Table S13. Ordinal regression results for predicting the functional assessment scale scores of patients with a LoF dominant negative variant compared to patients with a dominant negative variant, LoF or LoF presumed variant.

		95% HDI			
	Est.	lw. bound	up. bound	*BF*	*pd*
Functional Scales					
VSS	-0.46	-2.34	1.42	2.2	0.686
FCCS	-0.81	-2.52	0.81	5.1	0.837
GMFCS	0.05	-1.08	1.22	0.9	0.534
VFCS	-0.08	-1.29	1.13	1.2	0.551
EDACS	-0.41	-1.91	1.17	2.4	0.703

Note: GMFCS – Gross Motor Function Classification System; EADCS – Eating and Drinking Ability Classification System; FCCS – Functional Communication Classification System; VFCS – Visual Function Classification System; VSS – Viking speech scale

Table S14. Censored regression results for predicting age at indepedent walking based on variant type (age in months).

	Est.	95% HDI lw. bound	95% HDI up. bound	*BF*	*pd*
Missense variant	27.7	18.4	42.5		
Nonsense variant	41.0	35.2	47.0	15.21	0.938
Frameshift	39.2	33.3	45.2	11.00	0.917
Whole gene deletion	45.7	27.1	79.7	16.21	0.942
Splice	35.7	25.0	50.9	4.60	0.821

Table S15. Regression results for predicting adaptive skills based on variant type.

	Est.	95% HDI lw. bound	95% HDI up. bound	*BF*	*pd*
GAC					
Missense variant	76.9	51.8	100.8		
Nonsense variant	52.9	36.1	63.5	89.91	0.989
Frameshift	46.7	29.4	58.4	391.16	0.997
Whole gene deletion	61.5	31.5	88.7	3.77	0.79
Splice	53.9	32.5	71.6	21.25	0.955
Conceptual Composite					
Missense variant	90.2	75.1	101		
Nonsense variant	63.3	59.8	66.8	665.67	0.999
Frameshift	63.8	60.4	67.1	464.12	0.998
Whole gene deletion	58.5	31.5	72.2	407.16	0.998
Splice	61.5	48.2	70.3	376.36	0.997
Social Composite					
Missense variant	87	72	98.6		
Nonsense variant	66.8	63.4	70.5	79.97	0.988
Frameshift	64.1	59.5	67.9	185.92	0.995
Whole gene deletion	66.5	46.9	79.5	39.73	0.975
Splice	63.5	51.1	73.4	111.36	0.991
Practical Composite					
Missense variant	71.9	42.1	98.3		

Nonsense variant	51.3	34	64.1	18.23	0.948
Frameshift	40.5	22.1	55.9	121.7	0.992
Whole gene deletion	72.1	38.3	102.9	0.95	0.487
Splice	56.8	33.7	75.6	4.32	0.812

Table S16. Regression results for predicting symptoms of psychopathology based on variant type.

		95% HDI			
	Est.	lw. bound	up. bound	*BF*	*pd*
Anxious/Depressed					
Missense variant	56.6	47.5	62.3		
Nonsense variant	59.8	58	61.8	6.17	0.861
Frameshift	60.9	59.1	62.8	16.11	0.942
Whole gene deletion	56.2	43.1	64.3	0.96	0.489
Splice	62.4	57.3	67.1	16.59	0.943
Withdrawn Behavi					
Missense variant	67.7	56.5	77.2		
Nonsense variant	64.4	61.8	67.2	0.41	0.29
Frameshift	65.5	62.9	68.1	0.55	0.354
Whole gene deletion	62.5	48.6	74.5	0.36	0.265
Splice	66.9	58.9	74.9	0.87	0.467
Somatic Complaints					
Missense variant	64.0	55.9	69.8		
Nonsense variant	58.6	57.0	60.3	0.12	0.111
Frameshift	58.7	57.2	60.4	0.13	0.117
Whole gene deletion	54.6	42.6	61.7	0.05	0.052
Splice	61.8	56.6	65.6	0.43	0.298
Thought Problems					
Missense variant	73.4	57.8	88.9		
Nonsense variant	72.1	68.7	75.8	0.78	0.439
Frameshift	76.2	72.2	80.3	1.69	0.628
Whole gene deletion	51.7	35.4	68.2	0.03	0.029
Splice	72.5	62.7	82.0	0.86	0.462
Autism Spectrum					
Missense variant	67.5	55.6	80.4		

Nonsense variant	66.3	62.6	69.9	0.73	0.422
Frameshift	68.6	65.6	71.6	1.27	0.56
Whole gene deletion	67.5	51.2	87.1	1.00	0.501
Splice	79.2	68.7	89.2	11.11	0.917
Attention Problems					
Missense variant	67.6	57.1	77.8		
Nonsense variant	68.5	65.7	71.3	1.29	0.564
Frameshift	69.5	66.8	72.1	1.73	0.634
Whole gene deletion	63.6	49.7	75.6	0.45	0.312
Splice	71.8	64.2	79.1	2.89	0.743
Aggressive Behavior					
Missense variant	60.9	50.9	68.6		
Nonsense variant	62.2	59.8	64.6	1.68	0.627
Frameshift	63.3	61.1	65.8	2.79	0.736
Whole gene deletion	58.0	43.9	66.7	0.48	0.326
Splice	67.7	60.3	73.8	10.37	0.912

Table S17. Ordinal regression results for predicting scores on functional assessment scales based on variant type (missense variants vs. nonsense variants, frameshift variants, whole gene deletions and splice variants).

		95% HDI			
	Est.	lw. bound	up. bound	*BF*	*pd*
VSS					
Nonsense variant	1.94	-0.69	4.74	12.6	0.927
Frameshift	2.36	-0.2	5.20	25.7	0.963
Whole gene deletion	3.44	-0.28	7.54	29.5	0.967
Splice	1.42	-1.47	4.52	4.9	0.831
FCCS					
Nonsense variant	1.50	-0.96	3.96	8.5	0.895
Frameshift	1.24	-1.24	3.62	5.6	0.849
Whole gene deletion	5.00	1.01	9.01	195.1	0.995
Splice	0.77	-2.1	3.61	2.4	0.706
GMFCS					
Nonsense variant	5.35	1.50	10.94	>1000.00	>.999

Frameshift	5.89	2.10	11.59	>1000.00	>.999
Whole gene deletion	6.59	2.30	12.69	>1000.00	>.999
Splice	6.16	2.02	11.93	>1000.00	>.999
VFCS					
Nonsense variant	-0.47	-2.53	1.62	0.5	0.671
Frameshift	-0.50	-2.55	1.57	0.5	0.686
Whole gene deletion	-2.07	-5.67	1.23	0.1	0.896
Splice	0.47	-2.03	2.89	1.8	0.645
EDACS					
Nonsense variant	3.94	-0.19	10.14	56.3	0.983
Frameshift	4.22	0.02	10.33	95.6	0.990
Whole gene deletion	5.48	0.54	11.97	123.2	0.992
Splice	4.99	0.51	11.32	157.7	0.994
