# Supplementary material for: Genotypic, functional, and phenotypic characterization in CTNNB1 neurodevelopmental syndrome
Source: HGG Adv. 2025 Jul 18;6(4):100483. doi: 10.1016/j.xhgg.2025.100483 (PMC12335994; doi:10.1016/j.xhgg.2025.100483)
Supplement: Document S2. Article plus supplemental information [file mmc5.pdf]

HGG
Advances

ARTICLE

Genotypic, functional, and phenotypic characterization in CTNNB1 neurodevelopmental syndrome

Nina Žakelj,[1,15] David Gosar,[1,15] Špela Miroševič,[2] Stephan J. Sanders,[3,4,5] Alicia Ljungdahl,[3,4] Sayeh Kohani,[3] Shouhe Huang,[6] Lok I Leong,[6] Ying An,[6] Miou-Jing Teo,[6] Fiona Moultrie,[7] Roman Jerala,[8,9] Duško Lainšček,[8,9] Vida Forstnerič,[8] Petra Sušjan,[8] Leszek Lisowski,[10,11,12] Andrea Perez-Iturralde,[10] Jasna Oražem Mrak,[1] Ho Yin Edwin Chan,[6,14] and Damjan Osredkar[1,13,16,*]

Summary

CTNNB1 neurodevelopmental syndrome is a rare disorder caused by *de novo* heterozygous variants in the *CTNNB1* gene encoding β-catenin. This study aimed to characterize genetic variants in individuals with CTNNB1 neurodevelopmental syndrome, systematically assess the spectrum of clinical phenotypes using standardized measures, and explore potential genotype-phenotype correlations. In this cross-sectional cohort study, individuals diagnosed with CTNNB1 neurodevelopmental syndrome underwent structured interviews using standardized scales to evaluate motor skills, speech, communication, feeding abilities, visual function, neurodevelopment, and psychopathology. Genetic variants were analyzed, and, in a subset of cases, the impact of β-catenin variants on the Wnt/β-catenin signaling pathway was assessed. Across the 127 included participants (mean age, 70 months; range, 7–242 months) from 20 countries, we identified 88 different variants of the *CTNNB1* gene, 87 of which were predicted to lead to loss of *CTNNB1* function. Functional assays demonstrated reduced Wnt signaling activity, including 11 variants that also exhibited a dominant-negative effect. One missense variant demonstrated a gain-of-function effect. Dominant-negative variants were not clearly associated with a distinct phenotype; however, those with missense variants presented a milder phenotype, including earlier achievement of independent walking, fewer motor impairments, better conceptual and social skills, improved communication, and fewer feeding difficulties. This study describes the genetic, functional, and phenotypic characteristics in individuals with CTNNB1 neurodevelopmental syndrome. Further investigation into the genotypic and phenotypic characteristics of this syndrome and their interrelationships is essential to deepen our understanding of the disorder and inform the development of targeted therapies.

Introduction

β-catenin is an evolutionarily conserved protein that exerts a crucial role in a multitude of developmental and homeostatic processes in animals.[1,2] During embryonic development, Wnt-regulated β-catenin plays a critical role in establishing the body axis and the orchestration of tissue and organ development. In adult organs, Wnt signaling continues to play indispensable roles in tissue homeostasis, cell renewal, and regeneration.[1] Wnt signaling includes a canonical pathway, often referred to as the Wnt/β-catenin pathway, that involves the nuclear translocation of β-catenin and activation of target genes primarily to control cell proliferation and a non-canonical pathway that regulates cell polarity and migration. Wnt signaling is highly regulated, including mutual regulation of the canonical and non-canonical pathways,[3] and dysregulation is associated with serious outcomes, including cancer and neurodevelopmental disorders.[4,5]

CTNNB1 neurodevelopmental syndrome (MIM: 615075) is a rare disorder caused by germline *de novo* heterozygous variants in the *CTNNB1* gene encoding β-catenin (i.e., autosomal dominant). Approximately 400 individuals have been diagnosed with this syndrome worldwide,[6] with an estimated incidence of about 2.6–3.2 in 100,000 live births.[7] Due to the syndrome's rarity, insufficient phenotypic characterization, and limited medical awareness, CTNNB1 neurodevelopmental syndrome is likely underdiagnosed. Research indicates that at least one-quarter of individuals diagnosed with cerebral palsy (CP) have an

[1]Department of Pediatric Neurology, University Children's Hospital, University Medical Centre Ljubljana, Bohoričeva 20, 1525 Ljubljana, Slovenia; [2]Department of Family Medicine, Faculty of Medicine, University of Ljubljana, Ljubljana, Slovenia; [3]Institute of Developmental and Regenerative Medicine, Department of Paediatrics, University of Oxford, OX3 7TY Oxford, UK; [4]Department of Psychiatry and Behavioral Sciences, UCSF Weill Institute for Neurosciences, University of California, San Francisco, San Francisco, CA 94158, USA; [5]New York Genome Center, New York, NY 10013, USA; [6]School of Life Sciences, Faculty of Science, The Chinese University of Hong Kong, Hong Kong SAR, China; [7]MDUK Oxford Neuromuscular Centre, Department of Paediatrics, University of Oxford and NIHR Oxford Biomedical Research Center, OX3 9DU Oxford, UK; [8]Department of Synthetic Biology and Immunology, National Institute of Chemistry, Hajdrihova 19, 1000 Ljubljana, Slovenia; [9]Centre for Technologies of Gene and Cell Therapy, National Institute of Chemistry, Hajdrihova 19, 1000 Ljubljana, Slovenia; [10]Translational Vectorology Research Unit, Children's Medical Research Institute, Faculty of Medicine and Health, The University of Sydney, Westmead, NSW 2145, Australia; [11]Australian Genome Therapeutics Centre, Children's Medical Research Institute and Sydney Children's Hospitals Network, Westmead, NSW 2145, Australia; [12]Laboratory of Molecular Oncology and Innovative Therapies, Military Institute of Medicine - National Research Institute, Warsaw, Poland; [13]Center for Developmental Neuroscience, Faculty of Medicine, University of Ljubljana, Ljubljana, Slovenia; [14]Gerald Choa Neuroscience Institute, The Chinese University of Hong Kong, Hong Kong SAR, China
[15]These authors contributed equally
[16]Lead contact
*Correspondence: damjan.osredkar@kclj.si
https://doi.org/10.1016/j.xhgg.2025.100483.
© 2025 The Author(s). Published by Elsevier Inc. on behalf of American Society of Human Genetics.
This is an open access article under the CC BY license (http://creativecommons.org/licenses/by/4.0/).

underlying monogenic etiology,[8] with *CTNNB1* variants accounting for up to 4% of these cases.[9,10] While data from more than 400 individuals have been reported to date, only small cohorts have undergone systematic phenotypic evaluation.[11–15] Recently, baseline data from an in-person natural history study were published, involving 32 persons living with CTNNB1 neurodevelopmental syndrome.[11] However, this study mainly involved English-speaking participants, and no genotype-phenotype correlations were assessed.

The aim of this study was to systematically characterize genetic variants in a cohort of individuals diagnosed with the CTNNB1 neurodevelopmental syndrome, evaluate the spectrum of clinical phenotypes using standardized measures, investigate potential genotype-phenotype correlations, and integrate functional genomic data to guide potential therapeutic strategies.[12]

Subjects and methods

This is a cross-sectional cohort study, performed between May 2021 and November 2022. The study was approved by the National Medical Ethics Committee of the Republic of Slovenia (0120-80/2021/4) and preregistered on ClinicalTrials.gov (NCT04812119).

Recruitment

A call for participation was broadcast through various social media support groups for individuals diagnosed with a CTNNB1 neurodevelopmental disorder. The inclusion criteria were a genetically confirmed pathogenic or likely pathogenic variant in the *CTNNB1* gene and a signed informed consent form by the participant or a parent or caregiver of the participant if the affected participant was unable to provide it. There were no age, nationality, language, or phenotypic restrictions for recruitment. Upon signing the consent form, respondents were asked to provide (1) a copy of the genetic report, (2) a copy of their head and/or spine magnetic resonance imaging (MRI) report (if available), and (3) a copy of their electroencephalogram (EEG) report (if available). Respondents were then invited to an online interview, for which a translator was provided if needed.

Data collection and management

Study data were collected and managed using Research Electronic Data Capture (REDCap) electronic data capture tools hosted at the University Medical Center Ljubljana's secure institutional server.[13]

Genetic data

Bulk tissue gene expression data from 176 *postmortem* human brain samples from the dorsolateral prefrontal cortex[14] were assessed at nucleotide resolution across development by calculating the median read count per base (Figure S1). These were represented as a track in the UCSC genome browser[15] to facilitate comparison across genomic regions. Gene expression for exon subdivisions (Table S1) based on GRCh38/GENCODEv39 were assessed using DEX-seq and plotted across developmental time (Figure S2A). The ratio of expression for pairs of exon subdivisions was assessed across development using a linear model (Figure S2B). More details on these methods for per exon gene expression have been described previously.[16]

Genomic data were extracted from clinical genetics reports collected. Genomic coordinates were treated as the gold standard; where not present, the cDNA and protein description of the variant were used to derive the genomic coordinates. Where necessary, coordinates were lifted over from GRCh37 to GRCh38 and all variants were annotated to the 3,661-bp MANE Select ENST00000349496.11, NM_001904.4 transcript from GENCODEv39 of the ENSG00000168036.18, *CTNNB1* gene. Mutability for each variant was estimated based on trinucleotide genomic sequence.[17] Protein structure and domain information were derived from the UniProt Feature viewer.[18]

Cell constructs used for the analysis of β-catenin protein levels in *CTNNB1* variants

Plasmids

FLAG-tagged wildtype (WT) and mutant *pcDNA3.1*(+)-*CTNNB1* constructs were synthesized by Genewiz (Suzhou, China). The 42 *CTNNB1* variants included in this study are p.Ser23LysfsTer27, p.Ser47GlufsTer3, p.Arg90Ter, p.Arg95Ter, p.Pro100ArgfsTer5, p.Ile140SerfsTer3, Gln193Ter, p.Ile231LeufsTer2, p.Leu294Ter, p.Ala317ValfsTer8, p.Gln322ProfsTer31, p.Val325-GlufsTer24, p.Met328GlufsTer24, p.Thr330AspfsTer23, p.Tyr333Ter, p. Ser352fsTer, p.Pro355GlnfsTer2, p.Gly367Ter, p.Leu385GlnfsTer9, p.Arg386GlnfsTer9, p.Val406PhefsTer9, p.Glu462SerfsTer10, p.Arg474Ter, p.Gln482Ter, p.Gly490AlafsTer33, p.Leu498PhefsTer32, p.Arg535Ter, p.Val564GlyfsTer7, p.Gly575Arg, p.Leu577Arg, p.Arg587Ter, p.Ile607LeufsTer7, p.Cys619LeufsTer2, p.Cys619Ter, p.Glu634Ter, p.Glu642ArgfsTer6, p.Tyr654Ter, p.Ser663ArgfsTer15, p.Tyr670Ter, p.Glu692Asp, p.Ala694LeufsTer41, and p.Ile700AsnfsTer14. The TOPFlash-luciferase and FOPFlash-luciferase were kind gifts from Professor Randall Moon (Addgene plasmids # 12456 and 12457). The TOPFlash-luciferase construct harbors seven consecutive WT β-catenin binding sites, while all β-catenin binding sites in the FOPFlash-luciferase construct are mutated.

Cell culture and transfection

The human neuroblastoma SK-N-MC cells (HTB-10TM, American Type Culture Collection, Manassas, VA, USA) were cultured in DMEM (11995065, Thermo Fisher Scientific, Waltham, MA, USA) supplemented with 10% fetal bovine serum (F7524, Sigma-Aldrich, St Louis, MO, USA) and 1% penicillin–streptomycin (15140122, Thermo Fisher Scientific). The SK-N-MC cells were transfected with plasmids using Lipofectamine 2000 (11668019, Thermo Fisher Scientific). Transfection was carried out according to the manufacturer's instructions.

Dual-luciferase reporter assay

The dual-luciferase reporter assay (E1910, Promega, Madison, WI, USA) was carried out as described previously.[1] The *pTK-RL Renilla* luciferase vector (E2241, Promega) was used as an internal control reporter construct for the normalization of transfection efficiency. Both firefly and *Renilla* luminescence were recorded on a Spark multimode microplate reader (Tecan, Morrisville, NC, USA). The relative luciferase activity was calculated by dividing the firefly luminescence reading by the *Renilla* luminescence reading.

Cellular thermal shift assay

SK-N-MC cells were seeded in a six-well plate at a cell density of 5×10^5 cells/well and grown until 70% confluency. Cells were transfected with 3.0 μg of plasmids using Lipofectamine 2000. At 18 h after transfection, cells were harvested, collected by

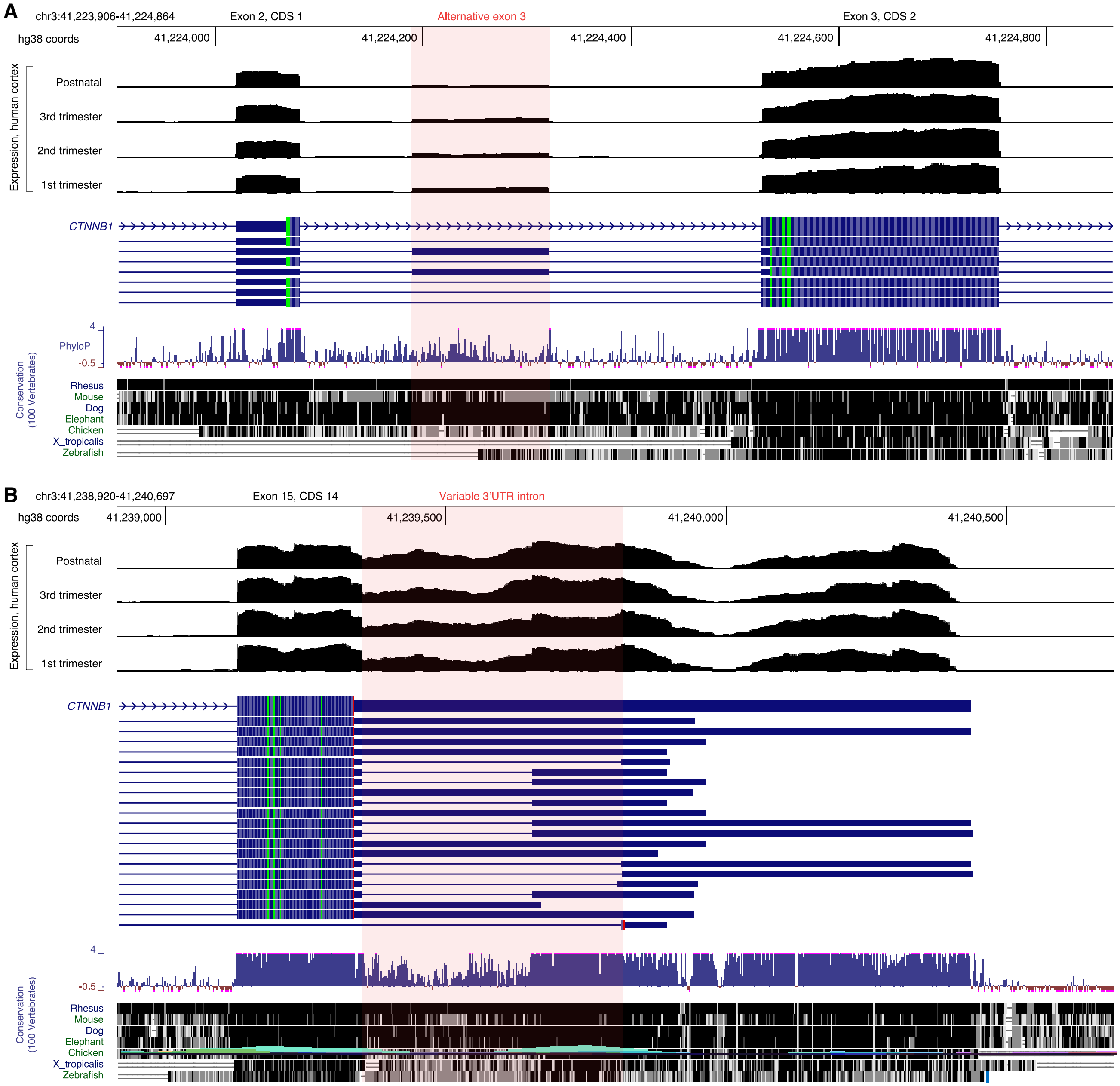

Figure 1. Alternative splicing in *CTNNB1*

(A) Genomic co-ordinates GRCh38/hg38 are shown at the top for the region around exons 2 and 3. Nucleotide resolution of the median read counts of RNA sequencing gene expression data from 176 *postmortem* human prefrontal cortices are shown organized by developmental stage; data are auto-scaled and include zero. A representative subset of protein-coding transcripts from GENCODEv46 are shown, the alternative splicing event of an additional 132 bp exon (chr3:41,224,190-41,224,321, hg38) is highlighted in light red. Species conservation across 100 vertebrates is represented at the bottom, including PhyloP scores (top) and homology across species (bottom).

(B) Corresponding data for an intron with variable inclusion in the 3′ UTR. Images were generated from UCSC genome browser.

centrifugation (200×*g*, 3 min) and washed with PBS twice. The whole cell pellet was resuspended in 800 μL of PBS and divided into 14 equal aliquots. The cell suspensions were incubated at a temperature range of 40.0°C, 42.3°C, 44.7°C, 47.0°C, 48.7°C, 50.7°C, 53.3°C, 55.2°C, 57.0°C, 60.0°C, 64.0°C, and 68.0°C for 6 min using a C1000 Touch Thermal Cycler (Bio-Rad Laboratories, Hercules, CA, USA), followed by cooling to room temperature for 6 min. One aliquot was kept at room temperature and one aliquot was kept on ice as controls. The samples were then snap frozen in liquid nitrogen for 1 min, with a total of three freeze-and-thaw cycles. Subsequently, the samples were briefly vortexed and centrifuged at 16,100×*g* for 25 min at 4°C to pellet cell debris together with precipitated and aggregated proteins. The soluble supernatant fraction was collected for western blot analysis. The protein signal was detected using the anti-FLAG antibody (1:1,000; F3165, Sigma-Aldrich). The band intensities were quantified using ImageJ software and normalized to the band intensity of the 40°C sample. The melting curves were fit

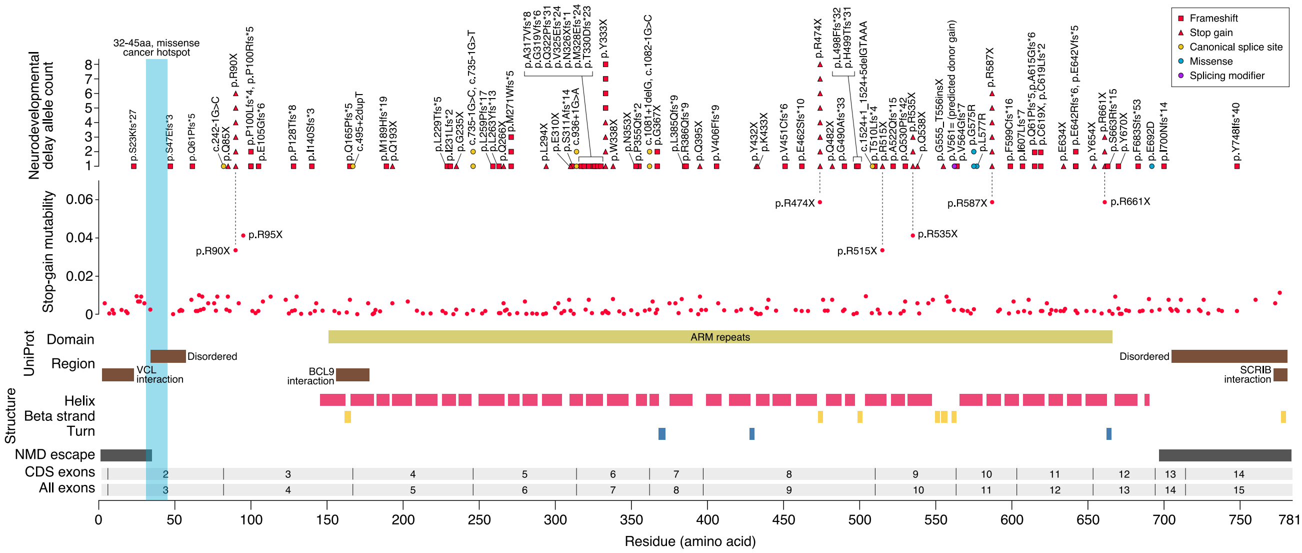

Figure 2. Neurodevelopmental disorder variants in *CTNNB1*

Variants reported by clinical genetics reports are annotated against the MANE Select transcript (ENST00000349496.11), the 125 variants are listed in Table S2. Predicted functional impact is indicated by shape and color (see legend). A blue bar indicates the region (p.32–45) frequently associated with somatic GoF mutations in cancer. For stop-gain single nucleotide variants, the predicted mutability based on trinucleotide genomic sequence is shown under the variants; predicted highly mutable stop gain sites are labeled. Below, protein domains, regions, and structure from UniProt and AlphaMissense are shown. At the bottom, the exon number, CDS exon number, and regions predicted to escape nonsense-mediated decay (NMD) are shown with protein residue number.
Nine variants disrupt the canonical splice site, and all of these are predicted to impact splicing (SpliceAI score ≥0.8). Two additional variants had high SpliceAI scores, suggesting they act as cryptic splice sites: the missense variant p.E692D (SpliceAI donor loss score 0.94, donor gain score 0.78) and the synonymous variant p.V561= (SpliceAI donor loss score 0.41, donor gain score 0.76).

by non-linear regression using the Boltzmann sigmoidal equation in GraphPad Prism 7 to determine the apparent melting (unfolding) temperature (Tm).

Immunoblotting

Protein samples were harvested from SK-N-MC cells using the SDS sample buffer (100 mM Tris-HCl, pH 6.8, 2% SDS, 40% glycerol, 5% β-mercaptoethanol, and 0.1% bromophenol blue). Samples were heated at 99°C for 10 min before being subjected to the immunoblotting analysis. The protein samples were then transferred to a PVDF membrane (IPVH00010, pore size 0.45 μm, Merck Millipore, Burlington, MA, USA). The membrane was blocked using 5% non-fat milk at 25°C for 1 h, followed by incubating primary antibodies at 4°C for 16 h. Primary antibodies used in this study were rabbit anti-FLAG (1:1,000; F3165, Sigma-Aldrich) and anti-β-tubulin (1:2,000, ab6046) from Abcam (Cambridge, UK).

The membrane was washed three times with 1× TBST each for 10 min before being subjected to the incubation of secondary antibodies at 25°C for 1 h. Secondary antibodies used were horseradish peroxidase (HRP)-conjugated goat anti-rabbit immunoglobulin (Ig)G (H + L) (11-035-045, 1:5,000) and HRP-conjugated goat anti-mouse IgG (H + L) (115-035-062, 1:10,000) from Jackson ImmunoResearch (West Grove, PA, USA). The membrane was washed three times with 1× TBST each for 10 min, followed by chemiluminescent signal detection. The signal was developed using Immobilon Forte Western HRP substrate (WBLUF0100, Merck Millipore), and the images were captured and processed using ChemiDoc Touch Imaging System. β-tubulin was used as the loading control. Only representative blots are shown.

Structured medical interview

All structured interviews were performed by a single physician-investigator (N.Ž.). Participants and/or their parents or caregivers were guided through a detailed medical questionnaire, containing more than 500 questions regarding medical history, functional status, and general demographic characteristics, including (1) family history of cancer, (2) detailed genetic information, (3) physical appearance data to ascertain potential dysmorphic features, (4) pregnancy and delivery data, (5) information on reaching developmental milestones and potential developmental delay, (6) first symptoms observed, (7) data on previous intellectual disability testing (if available), (8) current weight, height, and head circumference, (9) previous and current gait, muscle tone, and feeding status data, (10) data on potential neurological and cardiac symptoms, (11) potential presence of scoliosis, (12) head and/or spine MRI results (if available), (13) EEG results (if available) and potential abnormalities, (14) current list of medications, and (15) an array of standardized questionnaires on participant's behavioral, emotional, and functional status, as discussed further below.

Clinical instruments

An array of standardized tests was used to determine participant's behavioral, emotional and functional status. The tests were used in age groups pertaining to their respective standardization and validity.

(1) The Modified Checklist for Autism in Toddlers, Revised with Follow-Up: a parent-reported screening tool for autistic traits, standardized for children between 16 and 30 months of age.[19]

(2) The Autism Spectrum Quotient (AQ)-Children's Version: a parent-reported screening tool for autistic traits, standardized for children between 4 and 11 years of age.[20]

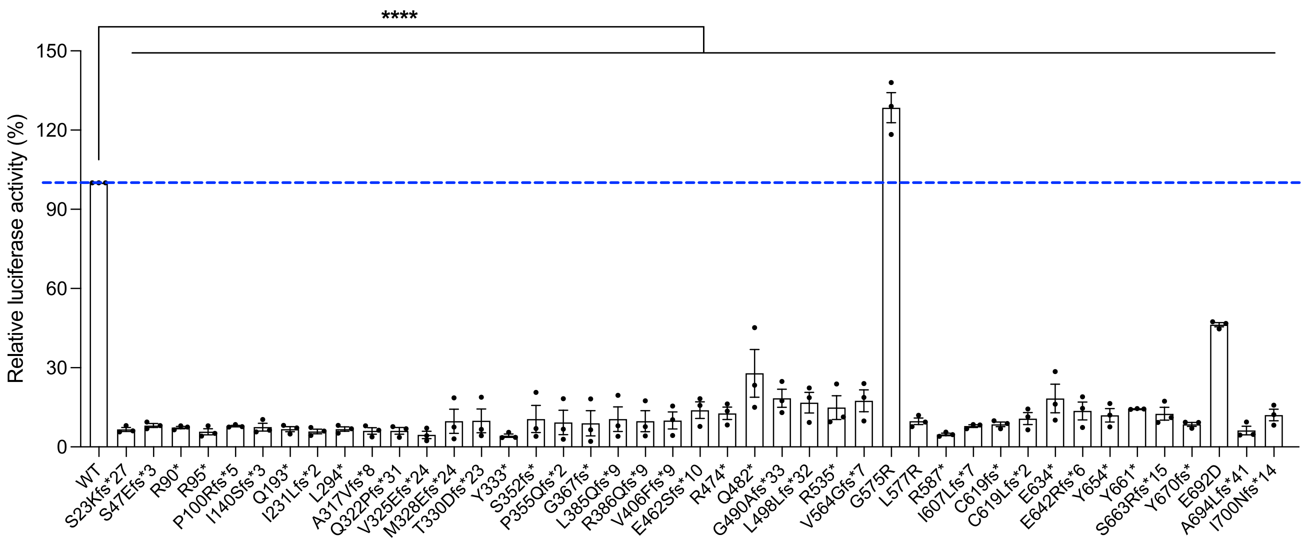

Figure 3. TOP/FOP Flash luciferase assay-based functional assessment of mutant β-catenin on Wnt transcriptional activity
Dual luciferase activity assay results from SK-N-MC cells transfected with $CTNNB1^{wt}$ or $CTNNB1^{mutant}$. Each bar indicates the relative luciferase activity for each construct following normalization to $CTNNB1^{wt}$ group. Results are from three independent experiments. Error bars denote SEM. ****$p < 0.0001$.

(3) The AQ-Adolescent Version: a self- or parent-reported screening tool for autistic traits, standardized for children above the age of 11 years.[21]
(4) Viking Speech Scale (VSS): a scale that classifies the speech performance of children with CP aged 4 years and older, incorporating the presence of a motor speech disorder and the severity of limitations in speech performance in everyday life.[22]
(5) Functional Communication Classification System: a scale that classifies how children with CP aged 4 years and older communicate with familiar and unfamiliar communication partners.[23]
(6) Brief Sleeping Questionnaire: a parent-reported screening test used to assess sleep patterns, parent perception, and sleep-related behaviors in children younger than 3 years.[24] Since sleep problems have previously been reported in children with CTNNB1 neurodevelopmental syndrome and the questionnaire is quite general, we decided to use it for all the children in our study.
(7) Pediatric Sleep Questionnaire (PSQ): a parent-reported screening test for sleep problems in children aged 2 years and older.[25]
(8) Eating and Drinking Ability Classification System (EDACS): a scale that classifies the ability to eat and drink safely and efficiently in children with CP aged 3 years and older.[26]
(9) Mini Manual Ability Classification System: a scale that classifies self-initiated ability to handle objects and the need for assistance or adaptation in daily activities in children with CP between 1 and 4 years of age.[27]
(10) Manual Ability Classification System: a scale that classifies self-initiated ability to handle objects and the need for assistance or adaptation in daily activities in children with CP between 4 and 18 years of age.[28]
(11) Gross Motor Function Classification System (GMFCS): a 5-level scale describing the gross motor function of children and youth with CP based on their self-initiated movement with emphasis on sitting, walking, and wheeled mobility.[29]
(12) Visual Function Classification System (VFCS): a five-level scale that describes how toddlers and youth (1–19 years) with CP use visual abilities in daily life.[30]
(13) Adaptive Behavior Assessment System (ABAS-3): a scale that assesses adaptive behavior and skills for people from birth through age 89.[31]
(14) The Achenbach System of Empirically Based Assessment (ASEBA): a scale for rating behavioral/emotional/social problems and adaptive characteristics for ages 1½ to more than 90 years.[32]

Please see the supplementary text for more precise description of the levels of the following scales: VSS, FCSS, GMFCS, VFCS, and EDACS.

Statistical analysis

Statistical analysis conducted in the R language for statistical computing[33] included the calculation of basic descriptive statistics for the demographic and phenotypical characteristics of participants. More specifically, for each phenotypical characteristic we analyzed the proportion of occurrence in our cohort with appropriate statistical confidence intervals (CIs) and/or calculated measures of central tendency and variability for continuous phenotypic measures (e.g., birthweight). We also calculated the proportions for each response category on measures of functioning in specific domains (VSS, FCSS, GMFCS, VFCS, and EDACS).

Finally, we used Bayesian regression implemented in the R *brms* package[34] to examine the phenotype-genotype correlation, i.e., the impact of *CTNNB1* variant type and *CTNNB1*-specific germline *de novo* variant on neurodevelopment. We examined the impact of the functional status of the *CTNNB1* gene (a) loss-of-function (LoF) dominant negative vs. LoF not dominant negative and (b) LoF dominant negative vs. LoF not dominant negative,

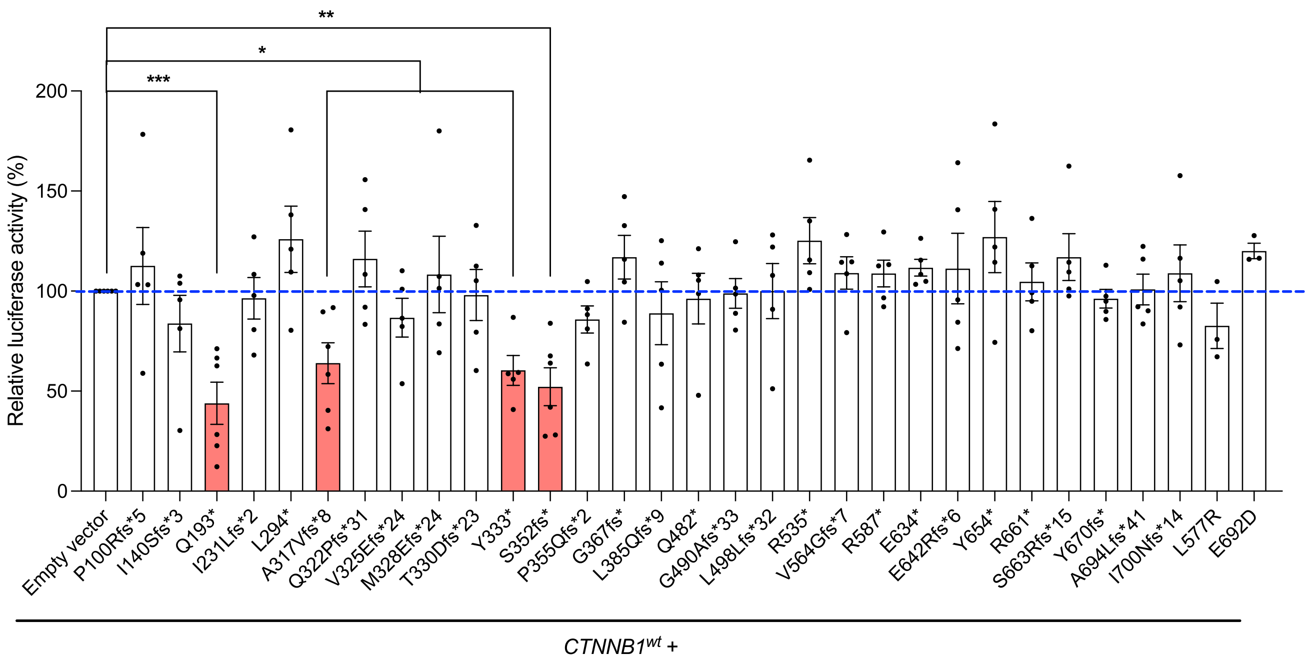

Figure 4. Study of dominant-negative effect of β-catenin variants on Wnt transcriptional activity using TOP/FOP Flash luciferase assay

Dual luciferase activity assay results from SK-N-MC cells co-transfected with *CTNNB1*^{wt} and empty vector (*pcDNA3.1*(+)) or *CTNNB1*^{mutant}. Each bar indicates the relative luciferase activity for each construct following normalization to the *CTNNB1*^{wt} (WT) + empty vector group. Variants marked in red are those for which a significant dominant-negative effect was observed. Results are from three to six independent experiments. Error bars denote SEM. *$p < 0.05$; **$p < 0.01$; ***$p < 0.001$.

LoF, and LoF presumed, as well as the variant type (missense vs. predicted haploinsufficiency, including frameshift, nonsense/stop gain, canonical splice site, and whole gene deletion) on the age at which individuals were able to walk independently using Bayesian survival models. We then examined the impact of variant type and variant functional impact on individuals' daily functioning as measured by the VSS, FCSS, GMFCS, VFCS, and EDACS by using Bayesian ordinal regression. To analyze differences between genotypes in their age of independent walking acquisition, we used Bayesian hurdle regression models. In the models, we included age at independent walking as the dependent variable and achievement of these milestones as the censoring variable. In addition to using genotype as a predictor, we also used the age of the child at entry into the study as a covariate in the hurdle part of the regression model. We examined the association of genotype with the presence of different neurological symptoms, adaptive skills, and symptoms of psychopathology using Bayesian regression with continuous response variables (ABAS-3, ASEBA, and age at independent walking) being modeled as skewed Gaussian distributions. For ABAS-3 adaptive skills scales, we used a regression analysis with a truncated response distribution, truncated at the lowest possible value of scale scores. Finally, binary response variables were modeled using Bayesian logistic regression. Each of the regression models was estimated using four separate Markov chain Monte Carlo (MCMC) chains and run for 10,000 iterations, with the last one-half being used for parameter estimation. We ensured that convergence was achieved for each model by checking the MCMC plots, *Rhat* statistic, and the effective sample size. We report the results of Bayesian analysis using both Bayes factors, regions of practical equivalence, as well as Bayesian equivalents of classical probability measures (*p* values).

We calculated Kaplan-Meier curves for the probability of achieving independent walking by using the R "survival" package.[35]

Results

Participants

Our study included 127 participants with a genetically confirmed pathogenic or likely pathogenic variant in the *CTNNB1* gene. Two additional participants whose genetic report indicated a variant in the *CTNNB1* gene, but without mentioning the specific variant, were not included. At the time of enrollment, the mean age of participants was 70 ± 50 months (range, 7–242 months) and 55 (43.3%) were female. The reporting individuals were mostly mothers (86.6%). Participants in the study were residents of 20 different countries: United States ($N = 28$), People Republic of China ($N = 26$), Spain ($N = 15$), Germany ($N = 12$), France ($N = 8$), Italy ($N = 7$), UK ($N = 6$), Poland ($N = 4$), Belgium ($N = 4$), Australia ($N = 3$), Mexico ($N = 2$), Portugal ($N = 2$), Russian Federation ($N = 2$), Switzerland ($N = 2$), Canada ($N = 1$), Ecuador ($N = 1$), Peru ($N = 1$), Slovenia ($N = 1$), Taiwan ($N = 1$), and Turkey (N = 1) (Figure S3). It is likely that several of these individuals were previously published in case reports or small cohorts.

Analysis of *CTNNB1* transcripts in the developing human brain

The MANE Select project has identified a canonical transcript for each gene based on multiple annotation features

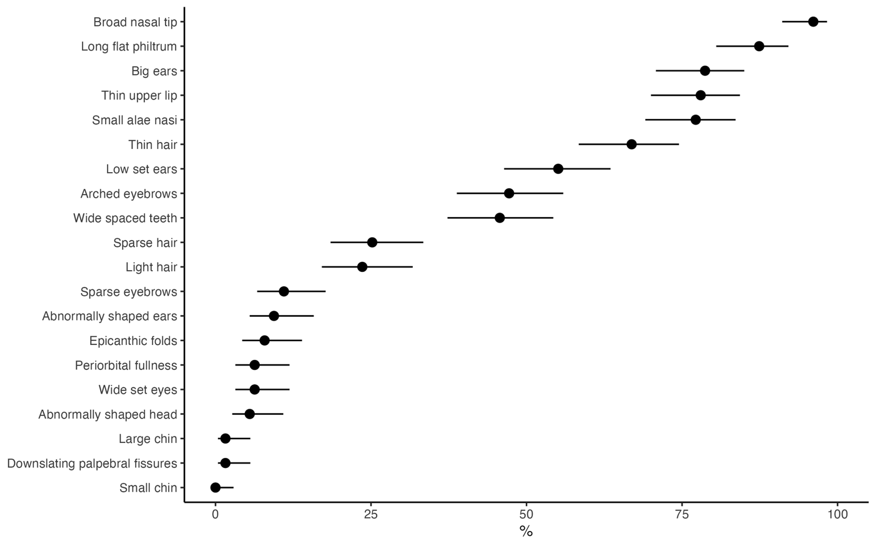

Figure 5. Prevalence of dysmorphic features in participants with CTNNB1 neurodevelopmental syndrome
Percentage with 95% CIs.

aligned between Ensembl/GENCODE and RefSeq.[36] Using bulk tissue gene expression data from 176 *postmortem* human prefrontal cortex samples across development, we assessed whether the MANE Select transcript was appropriate for a disorder with predominantly neurodevelopmental symptoms. These human cortex expression data support the use of the MANE Select transcript ENST00000349496.11, NM_001904.4 for this gene (Figure S1). We also assessed whether there were substantial differences in splicing patterns across human brain development; in contrast with other genes,[16] splicing remained mostly constant. The most developmental variability was observed in weakly expressed non-canonical transcripts (exon subdivisions 085 and 093 in Table S1; Figure S2).

We noticed three transcriptional features of interest (Figure S1). First, several alternative transcripts identified a transcription start site (TSS) upstream of the canonical TSS. We did not see evidence of this being expressed in the human prefrontal cortex but note that the genetic sequence at this locus is very highly conserved across species. This feature may represent a non-coding regulatory sequence related to *CTNNB1*. Second, multiple transcripts describe an additional 132-bp exon (alternative exon 3) between exons 2 and 3 in the MANE transcript that is predicted to lead to a β-catenin protein missing the first (N-terminus) five amino acids. We see evidence of this additional exon being expressed at moderate levels across the developing human cortex and the region is somewhat conserved across species (Figure 1A).[14] It is unclear if this additional exon impacts *CTNNB1* expression or β-catenin levels and function. Third, we see the variable inclusion of an intron in the 3′UTR of *CTNNB1* (Figure 1B, exon 16),[15] and this is supported by long-read RNA sequencing of the human cortex.[37] This event has previously been reported as being very frequently dysregulated in cancer,[38] furthermore, preventing inclusion of the intron reduced *CTNNB1* levels. This implies that promoting inclusion of this intron might lead to higher β-catenin levels, a potential therapeutic strategy in a haploinsufficient disorder.

Analysis of *CTNNB1* variants in neurodevelopmental disorders

We collected and curated 125 variants of the *CTNNB1* gene from the participant's clinical genetic reports (Table S2, including predicted protein impact and SpliceAI score). Of these, 91 (73%) were reported as pathogenic and 34 (27%) were reported as likely pathogenic. In 60 participants (48%), both parents also provided blood samples for genetic analysis; of these, all proband variants were found to be germline *de novo* mutations. Variants were annotated against the MANE Select transcript (Figure 2). The predominant pattern is of premature termination codons (PTCs), caused by frameshift variants, stop gain/nonsense, or canonical splice site variants. All three PTC mechanisms would be predicted to lead to nonsense-mediated decay and a haplo-insufficient (heterozygous LoF) mechanism of action, distinct from the gain-of-function (GoF) variants at the p.32-45 hotspot observed in cancer (Figure 2). Three PTCs (p.S23Kfs*27, p.I700Nfs*14, and p.Y748Ifs*40) were in regions predicted to escape nonsense-mediated decay (Figure 2).[18,39]

Only four missense variants were reported, giving a PTC: missense ratio of 29.5 (118:4). Similar ratios are observed in neurodevelopmental-associated genes in which PTCs cluster in the terminal exon (e.g., *PPM1D*, *ASXL3*) suggesting a toxic truncated protein (e.g., dominant negative)[40,41]; however, such clustering is not observed in *CTNNB1*. Among neurodevelopmental-associated genes with high PTC:missense ratios without PTC clustering (e.g., *ADNP*, *WAC*), the PTC enrichment in *CTNNB1* seems especially prominent. It is unclear whether this PTC enrichment represents patient ascertainment (e.g., missense variants lead to milder symptoms and less genetic testing or are not being reported as pathogenic) or mechanism (e.g., many missense variants cause more severe symptoms that are not compatible with life).

While the missense variant NM_001904.4:c.2076G>C is predicted to be benign at the protein level (p.(Glu692Asp)), *in silico* analysis using SpliceAI yielded a high score of 0.94, strongly suggesting a potential impact on pre-mRNA splicing. This raises the possibility that the variant could create or disrupt a splice site, leading to an altered transcript. Such an alteration might result in a frameshift and the introduction of a PTC, which would likely trigger

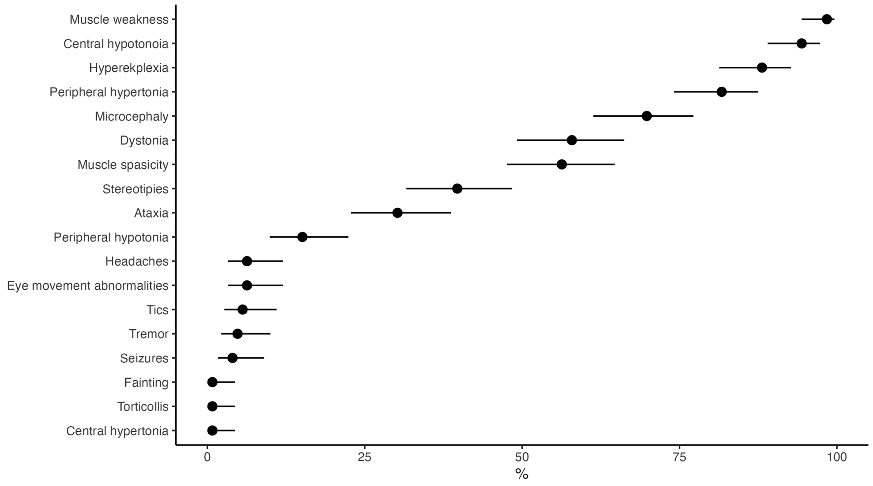

Figure 6. Neurological status of CTNNB1 participants as reported by parents
Percentage with 95% CIs.

nonsense-mediated mRNA decay and significantly reduce functional protein levels. Although experimental validation of this splicing effect was beyond the scope of the current study, the strong *in silico* evidence warrants the consideration of a potential LoF mechanism contributing to the observed phenotype, despite the benign missense prediction.

A recent analysis has raised the possibility that LoF variants in *CTNNB1* lead to a selective advantage in the male germline, as observed in achondroplasia. This mechanism would predict an older paternal age and a bias toward mutations on the paternal allele. Comparing the age of fathers at birth of the proband in this cohort (median, 33 years median; Table S2) with the Simons Simplex Collection (median, 32 years),[42] we do not see a difference ($p = 0.85$, Wilcoxon test, two-tailed). Estimation of parent of origin would require access to DNA samples for both parents and the child, which are not currently available.

Recurrent variants

We noted several PTC variants observed in multiple individuals (Figure 2). To understand these recurrent PTC variants, we assessed patterns of stop-gain mutability based on genomic sequence, driven by the 10-fold higher mutation rate of CpG variants.[17,43] We identified seven PTCs predicted to have higher mutation rates, and five of these aligned with observed recurrent PTCs: p.R474X ($n = 8$), p.R90X ($n = 6$), p.R587X ($n = 6$), p.R535X ($n = 3$), and p.R661X ($n = 3$), while p.R515X was observed only once and p.R95X was not observed at all. Surprisingly, eight individuals had a PTC predicted to lead to a stop gain at residue 333 (p.Y333X); however, the actual variant differed: c.998dupA ($n = 4$), c.999C>A ($n = 3$), and c.999C>G ($n = 1$). This recurrent variant cannot be explained by CpG mutability, homopolymers (recurrent nucleotides that enrich for indels), repetitive sequence, predicted splice sites, or predicted polyA sites.[44] This raises the possibility that the recurrence is driven by patient ascertainment, for example, a truncated protein with dominant negative effects leading to more severe symptoms. However, it is not clear why p.Y333X, but not other PTCs in *CTNNB1*, might act in such a manner. There is a high density of frameshift variants upstream of p.Y333X (e.g., residues 317–330) that might reflect a similar mutational or functional mechanism. Two individuals were reported to have the same missense variant (p.G575R), and this can also be explained by higher mutability driven by CpG (Table S3).

CTNNB1 variants can both perturb and stimulate the Wnt/β-catenin pathway

We used a luciferase-based reporter system (TOP/FOP Flash) to examine the molecular mechanism of the disease activity of the Wnt/β-catenin pathway in SK-N-MC cells overexpressing 43 mutant β-catenin constructs observed in persons living with the CTNNB1 syndrome. The TOPFlash- and FOPFlash-luciferase constructs are commonly used to evaluate β-catenin-dependent signaling, which drives the expression of TCF. When compared with the WT *CTNNB1*-transfected cells, cells expressing all *CTNNB1* variants, except for one, showed reduced levels of TOPFlash-luciferase activity. This strongly suggests a LoF mechanism for these *CTNNB1* variants, in keeping with nonsense-mediated decay predictions and functional loss of an allele. Intriguingly, cells transfected with the $CTNNB1^{G575R}$ missense variant showed a significantly higher TOPFlash-luciferase activity. This finding indicates that, unlike the other Wnt/β-catenin LoF variants, the $CTNNB1^{G575R}$ variant might act via stimulating the Wnt/β-catenin pathway (Figure 3); however, more studies are needed in other cell types.

Missense variants can lead to normal protein levels, as was observed for the variant p.G575R, or reduced protein levels, e.g., by inducing protein instability, as was observed for the variant p.L577R. In contrast, all PTC variants are predicted to induce nonsense-mediated decay and minimal protein levels, unless they occur 50 bp downstream of the last exon splice junction (Figure 2) or in the first 100 bp from the first codon. Of the 29 PTC variants assessed, 19 follow this prediction (Table S4; Figure S4). Fifteen PTC variants (p.L294*, p.T330Dfs*23, p.P355Qfs*2, p.R386Qfs*9, p.Q482*, p.G490Afs*33, p.L498Lfs*32, p.R535*, p.V564Gfs*7, p.R587*, p.I607Lfs*7, p.C619fs*, p.E634*, p.S663Rfs*15, and p.A694Lfs*41) showed reduced β-catenin levels and a further five PTC

Table 1. Levels of functioning across domains of speech and language (VSS, FCCS), feeding (EADCS), motor function (GMFCS), and visual functioning (VFCS)

	Level 1 (%)	Level 2 (%)	Level 3 (%)	Level 4 (%)	Level 5 (%)
VSS	2.8	30.6	38.9	27.8	N/A
FCCS	8.3	31.9	22.2	33.3	4.2
GMFCS	17.3	38.6	29.1	11.8	3.1
VFCS	29.1	12.6	54.3	2.4	1.6
EDACS	42.4	34.8	17.4	4.3	1.1

FCCS, Functional Communication Classification System; N/A, not applicable. Note: Higher levels represent greater levels of impairment across all scales. For further details please see subsection clinical instruments.

variants (p.R90*, p.R95*, p.V406Ffs*9, p.E462Sfs*10, and C619fs*) led to undetectable protein levels. However, 10 PTC variants (p.I140Sfs*3, p.Q193*, p.I231Lfs*2, p.V325 Efs*24, p.M328Efs*24, p.Y333*, p.G367fs*, p.E642Rfs*6, p.Y654*, and p.Y670fs*) showed a similar protein level as the WT control.

CTNNB1 variants that exhibit a dominant-negative effect on the Wnt/β-catenin pathway

The presence of a truncated protein has the potential to lead to additional damaging effects, including a dominant negative mechanism. We therefore assessed the impact of 31 variants on TOPFlash-luciferase activity when co-transfected with WT *CTNNB1*. Most variants had similar activity to the co-transfection of WT *CTNNB1* and empty vector, consistent with a simple LoF mechanism. However, four variants (p.Y333*, p.Q193*, p.A317Vfs8*, and p.S352fs*) showed reduced levels of TOPFlash-luciferase activity suggesting a dominant negative mechanism (Figure 4).

Arginine substitution variants between ARM repeats 10 and 11 affect β-catenin protein stability in opposite directions

We identified two variants in the proximity (p.G575R and p.L577R) at the junction of ARM repeats 10 and 11. Both the glycine and leucine residues were mutated to arginine in individuals. Interestingly, these two neighboring arginine substitution variants result in a totally opposite effect on TOPFlash-luciferase activity (Figure 3). The p.G575R caused increased Wnt activity while the p.L577R resulted in reduced TOPFlash-luciferase activity.

We attempted to understand the opposite TOPFlash-luciferase pattern by looking into the biophysical stability of these mutant proteins. With a single point variant, proteins may undergo conformational changes or different interactions with cellular partners, which might subsequently affect their function. A cellular thermal shift assay (CETSA) was conducted to investigate whether the p. G575R and p.L577R variants have different thermal stability of the mutant proteins. CETSA is a well-established method used to determine cellular protein stability, since proteins typically unfold and aggregate when exposed to elevated temperatures. Proteins with greater stability are more resistant to temperature changes and have a higher Tm.[2,3] The Tm of the β-catenin WT protein was determined as 51.11 ± 0.80°C, meaning that 50% of the β-catenin WT protein remained soluble at this temperature. For p.G575R, the Tm was shifted to 55.32 ± 0.51°C, indicating that the p.G575R mutant protein is more stable than the WT protein and might be degraded less efficiently by the degradation complex. In contrast, the Tm of p.L577R was 46.02 ± 0.84°C, suggesting that the p.L577R mutant protein was less stable, which may be responsible for its loss of function (Figure S5).

Clinical phenotypes

Prenatal and perinatal characteristics

The mean gestational age at birth was 39 ± 1.8 weeks, with the youngest participant being born at 33 gestational weeks. The mean birthweight was 2,955 ± 497 g (range, 1,500–4,139 g). Several prenatal (Figure S6A) and perinatal (Figure S6B) characteristics were identified in our cohort: intrauterine growth restriction, microcephaly at birth, and feeding issues after birth. Perinatal birth complications were rare, as evidenced by the high Apgar scores at 1 and 5 min (median, 9 [interquartile range (IQR), 2] and 10 [IQR, 1], respectively).

Anthropomorphic measures

Compared with their peers, at the time of enrollment the participants tended to be of smaller stature, being 0.70 SD shorter (95% CI, 0.30–1.00), and weight, 0.80 SD lighter (95% CI, 0.50–1.00), according to World Health Organization *z*-scores for weight and height in children.[45]

Dysmorphic features

The most common dysmorphic features of the *CTNNB1* phenotype in our cohort were a broad nasal tip (96.1%; 95% CI, 91.1%–98.3%), a long flat philtrum (87.4%; 95% CI, 80.5%–92.1%), small alae nasi (77.2%; 95% CI, 69.1%–83.6%), a thin upper lip (78.0%; 95% CI, 70.0%–84.3%), large ears (78.7%; 95% CI, 70.8%–85.0%), and thin hair (66.9%; 95% CI, 58.4%–74.5%) (Figure 5), consistent with previous reports.[46]

Neurological signs and symptoms

By far the most prevalent neurological symptoms in participants were muscle weakness (98.4%; 95% CI, 94.4%–99.6%), central hypotonia (94.5%; 95% CI, 89.1%–97.3%), syndromic atypical hyperekplexia (88.2%; 95% CI, 81.4%–92.7%), and peripheral hypertonia (81.9%; 95% CI, 74.3%–87.6%) (Figure 6). Many parents also reported dystonia (57.5%; 95% CI, 48.8%–65.7%), spasticity (56.7%; 95% CI, 48.0%–65.0%), and peripheral hypotonia (15.0%; 95% CI, 9.8%–22.2%). Stereotypical movements were also relatively common, occurring in 40.2% of participants (95% CI, 32.0%–48.9%). Among other conditions diagnosed at the time of the participants' inclusion in the study were microcephaly (70.1%; 95% CI, 61.6%–77.4%; defined as −2 SD), headaches (6.3%; 95% CI, 3.2%–11.9%), and seizures (3.9%; 95% CI, 1.7%–8.9%).

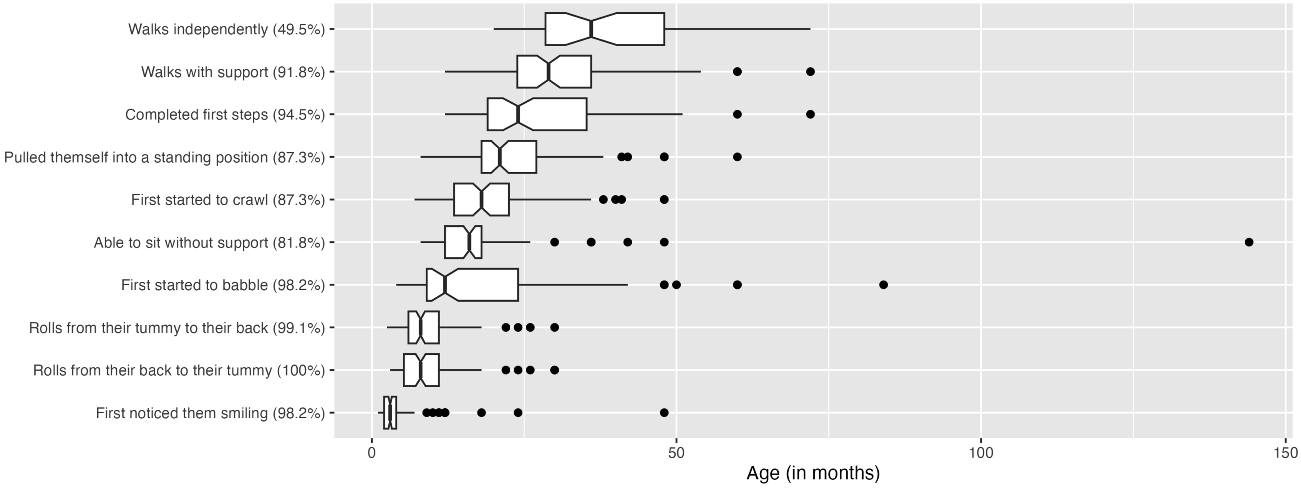

Figure 7. Developmental milestones of CTNNB1 neurodevelopmental syndrome participants aged 24 months or older ($N = 111$)
Percentages represent the proportion of participants achieving a particular milestone. Boxplots represent the median and IQR. Dots represent outliers.

Neurophysiological studies

EEG was performed in 81 of the 127 participants (63.8%). Most participants had a normal EEG, although seven (8.6%; 95% CI, 4.2%–16.8%) participants had an abnormal EEG. In two participants, an abnormal slowing of the background activity was reported, while in five participants, epileptiform discharges were reported. Five participants reported taking anti-seizure medications (valproic acid, phenobarbital, levetiracetam, oxcarbazepine, or clobazam, with some more taking than one).

MRI studies

MRI was performed in 121 of the 127 participants (95.3%). Most participants had a normal MRI, while 34 (28.1%; 95% CI, 20.9%–36.7%) had abnormal MRI findings. White matter abnormalities were reported in 26 of these 34 participants (76.5%). Among these, 15 participants were reported to have white matter volume loss, 11 participants had abnormal signal intensity, and 8 had delayed myelinization. Of these 26 participants, 5 also had an abnormal corpus callosum (CC) morphology, accompanied with a smaller CC volume in 4 participants. Gray matter abnormalities were specifically reported in three participants, while seven participants presented with wider cortical cerebrospinal fluid spaces.

Functional assessment

Assessment of participants' speech, language, feeding, motor and visual function using standardized scales is presented in Table 1.

Visual function

Another major area of reported symptomology were ophthalmologic issues (Figure S7). Most of the participants had strabismus (81.1%; 95% CI, 73.4%–87.0%) and 47.2% had farsightedness (95% CI, 38.7%–55.9%). Less common problems were near-sightedness (16.5%; 95% CI, 11.1%–24.0%), familial exudative vitreoretinopathy (FEVR; 7.1%; 95% CI, 3.8%–12.9%), and gaze palsy (3.1%; 95% CI, 1.2%–7.8%).

Motor development

The age at which CTNNB1 participants achieved their developmental milestones in the motor domain was significantly delayed (Figure 7). Considering participants older than 24 months, 91.9% were (95% CI, 85.2%–95.6%) able to walk with support with the average age of attaining this milestone being 30 months. Far fewer were able to walk independently (49.5%; 95% CI, 40.4%–58.7%; walked without aid for at least 10 m), with those that did achieve this milestone doing so at 39 months of age on average. Most individuals used a wheelchair in their daily life (72.7%; 95% CI, 59.8%–82.7%).

Looking more specifically at walking in our cohort of persons living with the CTNNB1 syndrome, up to 28.6% are not able to walk independently in any age group (Table 2). The probability of independent walking increases with advancing age (Figure 8).

Neurodevelopment

On the ABAS-3 (Figure S8), the majority of participants' scores were in the range of children and adolescents with mild (50.8%; 95% CI, 41.8%–59.9%) or moderate to severe intellectual disability (26.3%; 95% CI, 19.0%–35.1%). A minority achieved scores observed in neurotypical participants (3.5%; 95% CI, 1.3%–8.7%) or participants with borderline intellectual abilities (19.2%; 95% CI, 13.1%–27.5%). Among those CTNNB1 individuals who had already been psychologically assessed before participating in the study (40.9%), more than three-quarters had been given a diagnosis of intellectual disability (78.8%; 95% CI, 66.0%–97.8%).

Among the adaptive subdomains in which the participants had the greatest difficulties were subdomains included in the practical composite score (self-care, health and safety, and home living), as well as the communication subdomain. In concordance with the latter, we found that 38.9% (95% CI, 28.5%–50.4%) had unclear speech not usually understandable to unfamiliar listeners and

Table 2. Number and percentage of persons living with the CTNNB1 syndrome walking independently across age groups

	No.		%	
Age, mo	No	Yes	No	Yes
≤24	16	0	100.0	0.0
25–60	36	21	63.2	36.8
61–120	12	17	41.4	58.6
121–180	6	15	28.6	71.4
≥181	2	1	66.7	33.3

27.8% (95% CI, 18.8%–39.0%) had no understandable speech (Table S5).

On the ABAS-3, participants also displayed substantial impairments in pre-academic and academic skills. We found that, among the children who attended school or kindergarten (62.2%; 95% CI, 53.3%%–70.2%), most required additional support in school (92.7%; 95% CI, 80.6%%–97.5%) and one-half attended a special education kindergarten or school (48.1%; 95% CI, 37.4%–58.9%).

Before entering the study, most participants had not had a formal psychological assessment. However, among those who did, approximately one-half had been diagnosed with autism spectrum disorder (ASD; 48.3%; 95% CI, 39.6%–57.2%). This group of individuals also scored beyond the critical value for ASD on the autism spectrum scale of the Child Behavior Checklist 1½ 1–5 (mean, 72.7 ± 8.3) and higher than the remaining participants (mean, 65.3 ± 10.4). Among those diagnosed with ASD, 57.9% also had a diagnosis of intellectual disability (95% CI, 36.3%–76.9%), while among those without the diagnosis, 36.4% had a diagnosis of intellectual disability (95% CI, 19.7%–57.0%)

In our cohort, other psychopathological symptoms were also more common than in the general population. On the ASEBA scales, respondents reported increased rates of withdrawn behavior, symptoms of anxiety and depression, attention problems, and thought problems. Symptoms of ASD, as assessed with the ASEBA preschool scales, were also more prevalent, with about half of the participants scoring above the clinical cut-off of a T-score of 70.

Developmental regression

A substantial proportion (35.1%; 95% CI, 26.9%–44.4%) of respondents reported signs of developmental regression in participant's ability to talk or babble (19.8%; 95% CI, 13.5%–28.2%), and walk independently (5.4%; 95% CI, 2.5%–11.3%). When present, most respondents noticed the regression at 31 ± 40 months of age (range, 3–180 months).

Parents also reported that 56.3% of children living with the CTNNB1 syndrome (95% CI, 47.6%–64.7%) had sleeping problems. The average score on the PSQ was 7.1 ± 2.6 points (range, 2–15), with the established cut-off value suspected of obstructive sleep apnea being 7.3 points.[25]

Genotype-phenotype correlations

Genotype-phenotype correlations were examined by comparing motor development, neurodevelopment, and the functional status of individuals with (1) LoF dominant negative ($N = 11$) vs. LoF not dominant negative variant ($N = 38$), (2) LoF dominant negative variant ($N = 11$) vs. LoF not dominant negative ($N = 38$), LoF ($N = 24$), and LoF presumed variant ($N = 48$); and (3) different variant types (missense vs. predicted haploinsufficiency including frameshift, nonsense/stop gain, canonical splice site, and whole gene deletion).

LoF dominant-negative variants vs. LoF not-dominant-negative variants

We did not see evidence of differences between individuals with LoF dominant negative and individuals with LoF non-dominant variants in terms of age at which they were able to walk independently (LoF dominant negative, 36.0 months; LoF not dominant negative, 33.7 months; Bayes factor [*BF*], 1.65; Table S6), nor in terms of their adaptive functioning (*BF* values <2.1, *pd* values <0.678; Table S7), psychopathology (*BF* values <9.5, *pd* values <0.904; Table S8), or results of functional assessment (*BF* values <6.9, *pd* values <0.873; Table S9). The largest *BF* was from autism spectrum, with more symptoms reported in children with LoF not dominant negative ($BF = 9.46$, $pd = 0.904$).

LoF dominant-negative variants vs. LoF not-dominant-negative, LoF, and LoF presumed variants

Results were similar (Tables S10–S13) with the addition of 72 samples with variants predicted to be LoF and not known to have dominant negative effects (LoF nDN+). Of note, parents of individuals with the LoF nDN+ variants reported that these individuals had more symptoms of ASD compared with individuals with LoF dominant negative variants ($BF = 26.9$, $pd = 0.964$).

Variant type

Clearer genotype-phenotype correlations emerged when looking at different variant types. We found plausible evidence that individuals with a missense variant ($n = 4$) started to walk earlier (median estimate, 27.7 months) compared with individuals with a nonsense (median estimate, 41.0 months) or frameshift variant (median estimate, 39.2 months) or a whole gene deletion (median estimate, 45.7 months; *BF* values >11.0, *pd* values >0.917; Table S14). Individuals with a missense variant were also rated as having better conceptual and social skills compared with individuals with other variant types (*BF* values >39.7, *pd* values >0.975; Table S15). In terms of psychopathology, individuals with a missense variant did not differ from other variant types, although their parents tended to report fewer symptoms of anxiety and depression compared with individuals with a frameshift variant ($BF = 16.11$, $pd = 0.924$) or splice variant ($BF = 16.59$, $pd = 0.943$); however, parents also tended to report more

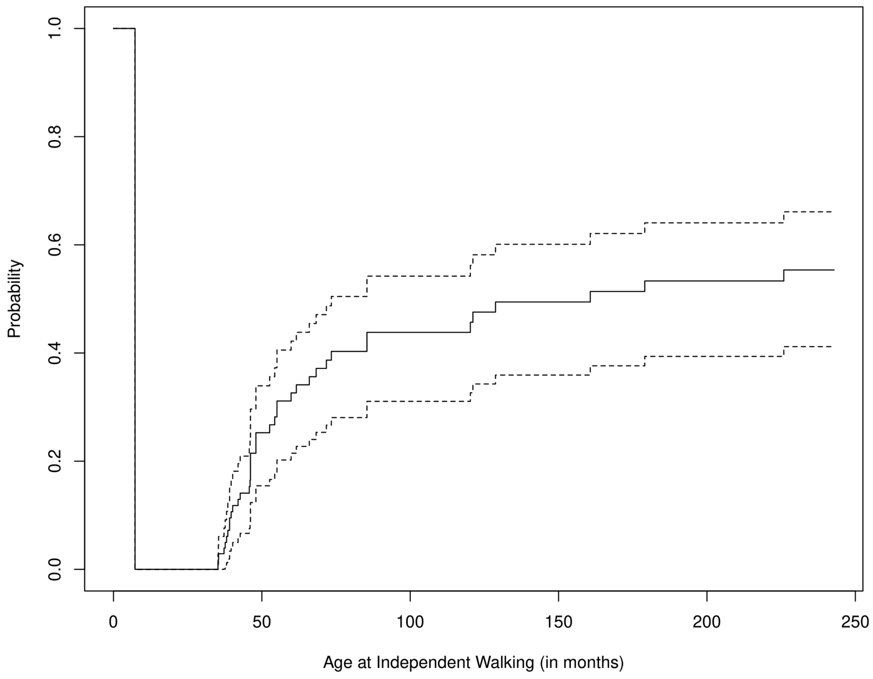

Figure 8. Kaplan-Meier curves showing the probability of independent walking by age with 95% CIs

somatic problems compared with individuals with a nonsense or frameshift variant or a whole gene deletion (*BF* values <0.13, *pd* values <0.117). In terms of psychopathology, the greatest symptom burden, compared with individuals with a missense variant, was present in individuals with a canonical splice variant. Their parents reported more pronounced symptoms of anxiety and depression ($BF = 16.59$, $pd = 0.943$), ASD ($BF = 11.11$, $pd = 0.917$), and aggressive behavior ($BF = 10.37$, $pd = 0.912$; Table S16).

On the functional assessment scales, individuals with a missense variant were rated as having better communication skills on the VSS compared with individuals with a nonsense variant, frameshift variant, or a whole gene deletion (*BF* values >12.6, *pd* values >0.927). They also showed the least difficulties in terms of feeding as rated by the EDACS (*BF* values >56.3, *pd* values >0.983). Finally, the analysis of GMFCS scores indicated better motor abilities than individuals with other variant types (*BF* values >1,000.0, *pd* values >0.999; Table S17; Figure 9).

Gain and loss of function

Given that only two individuals had a confirmed GoF variant in CTNNB1, we could only examine the genotype-phenotype correlation using descriptive means. When examining the age of onset for independent walking, as well as the median scores on measures of adaptive functioning and psychopathology, we found no evidence of greater impairment in individuals with a GoF variant. On the contrary, they showed better adaptive skills compared with the remaining individuals (Figure 10).

Discussion

We present the phenotypic spectrum of a large cohort of individuals living with the CTNNB1 neurodevelopmental syndrome and report possible genotype-phenotype correlations between missense and PTC variants. Rare diseases, like CTNNB1 neurodevelopmental syndrome, are often insufficiently characterized due to underdiagnosis, the rarity of identified individuals, and the lack of comprehensive disease registries that facilitate research. Additionally, research initiatives in the field of rare diseases are commonly limited. However, gaining a deeper understanding of the genetics, pathophysiology, and phenotypes of a specific rare disease is crucial for improving symptom management, developing treatment guidelines, and advancing the potential for disease-modifying therapies.[12]

Genetic characterization and Wnt transcriptional activity of *CTNNB1* variants

In our cohort of 125 participants with a genetically confirmed CTNNB1 neurodevelopmental syndrome, the predominant genetic pattern found was PTC, caused by frameshift variants, stop-gain, or canonical splice site variants. These variants were predicted to lead to nonsense-mediated decay and a haploinsufficient mechanism of action. Only four missense variants were reported (3.2% [4/125 variants]). Similarly, in the study of Kayumi et al.,[6] most *CTNNB1* variants (91.1% [357/392 variants]) were predicted to be LoF variants, predominantly stop gain/nonsense and frameshift variants, while missense variants accounted for only 7.1% of variants (28/392). This high PTC:missense ratio could represent that missense variants lead to milder symptoms postponing genetic testing in these participants or that missense variants cause more severe, life-limiting symptoms. Our data from four individuals with a missense variant supports the former explanation, with a milder phenotype compared with participants with other variants.

Our functional characterization of 43 variants supports the notion that haploinsufficiency, along with reduced Wnt activity, are the predominant pathological mechanisms. This suggests that strategies to increase gene expression, such as CRISPR activation or antisense oligonucleotides, could potentially be suitable therapies. The variable inclusion of an intron in the 3′ UTR of *CTNNB1*[38]

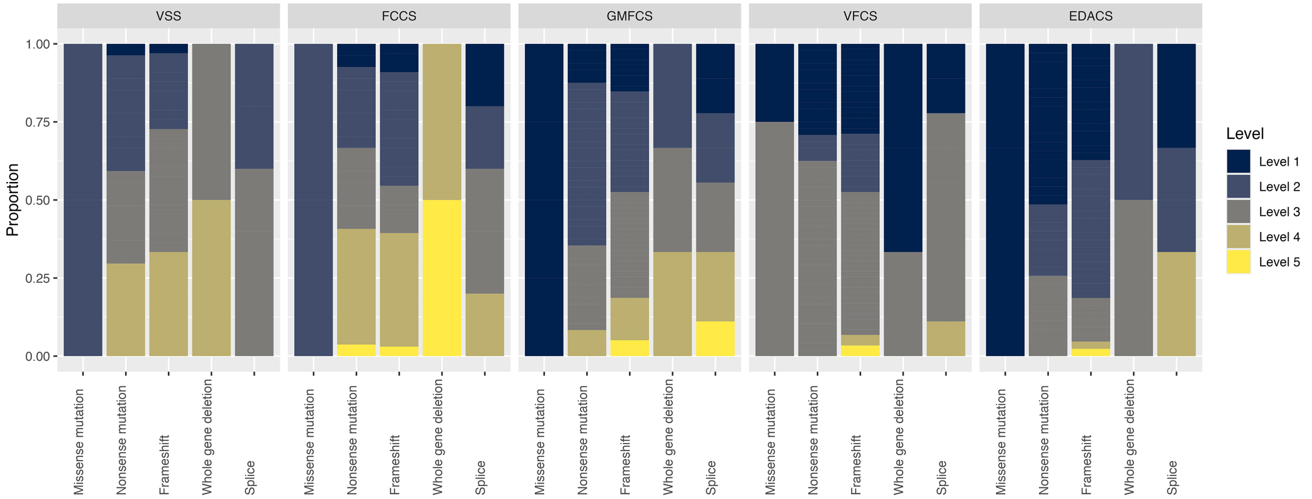

Figure 9. Scores on functional assessment scales by individuals in relation to their variant type (missense variants, nonsense variants, frameshift variants, whole gene deletions and (canonical) splice variants)
Level 1, milder phenotype; level 5, more severe phenotype.

(Figure 1B, exon 16), supported by long-read RNA sequencing of the human cortex,[37] presents one such therapeutic opportunity for increasing expression by promoting intron inclusion.

Surprisingly, the p.Y333* variant was observed in eight participants (four single nucleotide variations and four insertion/deletions), despite no there being evidence of genomic factors to account for this recurrence via mutational mechanisms. Participant ascertainment, driven by particularly severe symptoms in one or more domains, might explain this. Analysis of the cell construct showed that the variant did not lead to nonsense-mediated decay, as predicted, but rather a truncated protein. Co-expression with a WT construct suggested a dominant negative mechanism. The p.Q193*, p.A317Vfs8*, and p.S352fs* variants were also found to result in a truncated protein and exert a dominant negative effect, although these variants were only observed in single participants. As a subgroup, we did not find evidence that variants with a dominant negative effect led to clear genotype-phenotype correlations that would phenotypically distinguish them from other LoF variants.

We also identified one missense variant that increased Wnt activity and was found to encode a more thermally stable protein, p.G575R (two participants in our cohort). This suggests a GoF effect. Although our cohort consisted of only two participants with this variant, descriptive observation suggests these participants were not different regarding the onset of independent walking, adaptive functioning, and psychopathology, but showed better adaptive skills compared with the remaining individuals.

These putative dominant-negative and GoF effects warrant further examination, since a therapy that would upregulate both *CTNNB1* alleles might have unintended consequences in individuals harboring these variants. Somatic missense variants of the *CTNNB1* gene in cancer patients also merit further investigation, as somatic GoF variants are functionally different from therapeutic overexpression, such as gene replacement.[47] In conclusion, genetic characterization of larger cohorts, combined with functional assessment of mutant β-catenin, would enable more precise genotype-phenotype analysis. This could better inform the development of targeted therapies and guide selection of treatment candidates once such treatments become available.

Typical phenotypic presentation

A significant proportion of participants experienced intrauterine growth restriction, with a low birth weight (<2,500 g), small head circumference, and postnatal feeding difficulties. At enrollment, many had reduced height and weight, microcephaly, and dysmorphic features (e.g., broad nasal tip, small alae nasi, long philtrum, thin upper lip, and large ears), observed in at least 75% of the cohort. Recognition of these features may help to decrease diagnostic delays in CTNNB1 syndrome.

Neurological findings were prominent, with more than 50% showing muscle weakness, hypotonia (central and peripheral), spasticity, dystonia, and atypical hyperekplexia—consistent with previous reports.[48] Motor disorders were often accompanied by developmental delay: 91.9% of children older than 24 months could walk with support, but only 49.5% achieved independent walking, often delayed. Wheelchair use was common (72.7%), and developmental regression was reported in 35.1%.

Participants frequently had speech and language impairments, feeding difficulties, visual issues (strabismus in 81.1%, while FEVR [7.1%] and gaze palsy [3.1%] were reported less commonly), and behavioral problems. Mild intellectual disability was found in more than 50%, while 26.3% had moderate to severe disability. ASD was reported in 48.3%, along with academic challenges and increased rates of anxiety, depression, attention deficits, and cognitive issues.

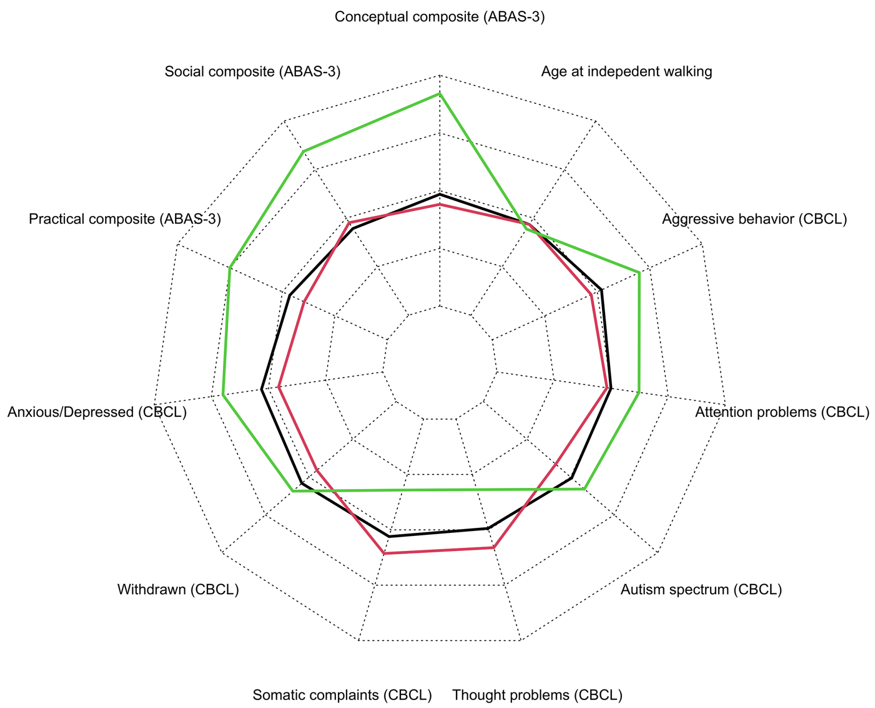

Figure 10. Adaptive skills, age at independent walking, and psychopathological symptoms for individuals with a confirmed GoF ($n = 2$; green), LoF ($n = 24$; red), or other variants ($n = 99$); please see the Table S2 for the list of specific variants included in these groups

Median standardized z-scores for each domain are shown (range for z-scores, −3.00 to 3.00) with higher scores indicating better functioning. CBCL, Child Behavior Check List.

MRI abnormalities were seen in 28.1% of participants, primarily nonspecific white matter changes (76.5% of those with findings). EEG abnormalities were rare, observed in only 8.6% of cases.

Genotype-phenotype correlations

As mentioned above, we were not able to find clear evidence that the 11 participants with dominant-negative LoF variants have specific phenotypes that would distinguish them from other participants. However, the four participants with a missense variant in the *CTNNB1* gene were more likely to start walking earlier compared with participants with other variants. Participants with a missense variant were also rated as having better conceptual and social skills compared with participants with other variants. On the functional assessment scales, participants with a missense variant were rated as having better communication skills and showed fewer difficulties in terms of feeding as rated by the EDACS. Finally, the analysis of GMFCS scores indicated that their motor abilities were clearly superior to those of participants with other types of variants. In terms of psychopathology, the greatest symptom burden was found in participants with a splice variant ($n = 9$), as their parents reported more pronounced symptoms of anxiety and depression, ASD and aggressive behavior.

Limitations

Although this is the largest cohort of CTNNB1 participants studied systematically with standardized tests, only a proportion of known individuals living with the CTNNB1 neurodevelopmental syndrome were enrolled. The number of participants was low for statistical purposes; therefore, more or different genotype-phenotype correlations could possibly be identified in larger cohorts of participants. The voluntary enrollment process through social media could also lead to selection bias, which could significantly skew the landscape of genetic variants and phenotypes described. Evaluating participants using standardized questionnaires and scales through online interviews—sometimes with a translator—provides an overview of their symptoms and enables an inclusive approach, allowing participation from individuals with diverse backgrounds and those living far from investigation centers. However, it cannot replace in-person examinations, such as psychological, ophthalmological, and neurological evaluations. Finally, there may be some overlap between the present cohorts and previously published cases. Due to data confidentiality, it was not possible to systematically assess overlap with previous publications, although we note that many of the individuals were based in Europe.

Suggestions for future studies

A detailed natural history study with in-person and longitudinal examinations using standardized measures would overcome many of the limitations mentioned above and would be essential for better understanding the CTNNB1 neurodevelopmental syndrome. Examinations such as MRI and wearable devices for movement analysis[49] could provide crucial additional data in understanding the syndrome. Identifying reliable blood biomarker(s) of the disease severity would be a significant aid in objectively assessing participants. Furthermore, such data would also provide important information for the design and development of potential disease modifying treatments.[12]

Conclusions

Although the CTNNB1 neurodevelopmental syndrome is a rare and understudied syndrome, rapidly expanding knowledge related to it is stimulating scientific and clinical inquiry. In the cohort of 125 participants described in this study, we have found that most carry a variant

leading to a loss of *CTNNB1* gene function and a reduction in Wnt signaling activity. However, we have identified a minority of variants with associated dominant negative or a GoF effect, warranting further studies of the genetic landscape of the CTNNB1 neurodevelopmental syndrome. This finding could be potentially important in the context of the development of potential disease modifying treatments.

Data and code availability

The majority of the data are provided in the manuscript and its supplementary materials. A reasonable request for R code should be addressed to the corresponding author.

Acknowledgments

We thank the persons living with CTNNB1 syndrome and their parents and caregivers for participating in the study. This work was supported by funding from the Slovenian Research and Innovation Agency (J7-4537 and P3-0458 to D.O.), from the National Institute of Mental Health (U01MH122681 and R01MH129751 to S.J.S.) and the UKRI Medical Research Council (MR/Z504725/1 to S.J.S.).

Declaration of interests

The authors declare no conflict of interest.

Supplemental information

Supplemental information can be found online at https://doi.org/10.1016/j.xhgg.2025.100483.

Received: January 24, 2025
Accepted: July 14, 2025

References

1. Valenta, T., Hausmann, G., and Basler, K. (2012). The many faces and functions of β-catenin. EMBO J. *31*, 2714–2736.
2. van der Wal, T., and van Amerongen, R. (2020). Walking the tight wire between cell adhesion and WNT signalling: a balancing act for β-catenin. Open Biol. *10*, 200267.
3. Liu, J., Xiao, Q., Xiao, J., Niu, C., Li, Y., Zhang, X., Zhou, Z., Shu, G., and Yin, G. (2022). Wnt/β-catenin signalling: function, biological mechanisms, and therapeutic opportunities. Signal Transduct. Target. Ther. *7*, 3.
4. AmeliMojarad, M., AmeliMojarad, M., Cui, X., and shariati, P. (2023). Pan-cancer analysis of CTNNB1 with potential as a therapeutic target for human tumorigenesis. Inform. Med. Unlocked *42*, 101331.
5. de Ligt, J., Willemsen, M.H., van Bon, B.W.M., Kleefstra, T., Yntema, H.G., Kroes, T., Vulto-van Silfhout, A.T., Koolen, D.A., de Vries, P., Gilissen, C., et al. (2012). Diagnostic exome sequencing in persons with severe intellectual disability. N. Engl. J. Med. *367*, 1921–1929.
6. Kayumi, S., Pérez-Jurado, L.A., Palomares, M., Rangu, S., Sheppard, S.E., Chung, W.K., Kruer, M.C., Kharbanda, M., Amor, D.J., McGillivray, G., et al. (2022). Genomic and phenotypic characterization of 404 individuals with neurodevelopmental disorders caused by CTNNB1 variants. Genet. Med. *24*, 2351–2366.
7. López-Rivera, J.A., Pérez-Palma, E., Symonds, J., Lindy, A.S., McKnight, D.A., Leu, C., Zuberi, S., Brunklaus, A., Møller, R. S., and Lal, D. (2020). A catalogue of new incidence estimates of monogenic neurodevelopmental disorders caused by de novo variants. Brain *143*, 1099–1105.
8. Chopra, M., Gable, D.L., Love-Nichols, J., Tsao, A., Rockowitz, S., Sliz, P., Barkoudah, E., Bastianelli, L., Coulter, D., Davidson, E., et al. (2022). Mendelian etiologies identified with whole exome sequencing in cerebral palsy. Ann. Clin. Transl. Neurol. *9*, 193–205.
9. Moreno-De-Luca, A., Millan, F., Pesacreta, D.R., Elloumi, H. Z., Oetjens, M.T., Teigen, C., Wain, K.E., Scuffins, J., Myers, S.M., Torene, R.I., et al. (2021). Molecular Diagnostic Yield of Exome Sequencing in Patients With Cerebral Palsy. JAMA *325*, 467–475.
10. Jin, S.C., Lewis, S.A., Bakhtiari, S., Zeng, X., Sierant, M.C., Shetty, S., Nordlie, S.M., Elie, A., Corbett, M.A., Norton, B. Y., et al. (2020). Mutations disrupting neuritogenesis genes confer risk for cerebral palsy. Nat. Genet. *52*, 1046–1056.
11. Sudnawa, K.K., Garber, A., Cohen, R., Calamia, S., Kanner, C. H., Montes, J., Bain, J.M., Fee, R.J., and Chung, W.K. (2024). Clinical phenotypic spectrum of CTNNB1 neurodevelopmental disorder. Clin. Genet. *105*, 523–532.
12. Miroševič, Š., Khandelwal, S., Amerson, E., Parks, E., Parks, M., Cochran, L., González Hernández, A., Ferraro, M., Lisowski, L., Perez-Iturralde, A., et al. (2025). Paving the way toward treatment solutions for CTNNB1 syndrome: a patient organization perspective. Ther. Adv. Rare Dis. *6*, 26330040251318355.
13. Harris, P.A., Taylor, R., Minor, B.L., Elliott, V., Fernandez, M., O'Neal, L., McLeod, L., Delacqua, G., Delacqua, F., Kirby, J., et al. (2019). The REDCap consortium: Building an international community of software platform partners. J. Biomed. Inform. *95*, 103208.
14. Werling, D.M., Pochareddy, S., Choi, J., An, J.Y., Sheppard, B., Peng, M., Li, Z., Dastmalchi, C., Santpere, G., Sousa, A. M.M., et al. (2020). Whole-Genome and RNA Sequencing Reveal Variation and Transcriptomic Coordination in the Developing Human Prefrontal Cortex. Cell Rep. *31*, 107489.
15. Nassar, L.R., Barber, G.P., Benet-Pagès, A., Casper, J., Clawson, H., Diekhans, M., Fischer, C., Gonzalez, J.N., Hinrichs, A.S., Lee, B.T., et al. (2023). The UCSC Genome Browser database: 2023 update. Nucleic Acids Res. *51*, D1188–D1195.
16. Liang, L., Fazel Darbandi, S., Pochareddy, S., Gulden, F.O., Gilson, M.C., Sheppard, B.K., Sahagun, A., An, J.Y., Werling, D.M., Rubenstein, J.L.R., et al. (2021). Developmental dynamics of voltage-gated sodium channel isoform expression in the human and mouse brain. Genome Med. *13*, 135.
17. Samocha, K.E., Robinson, E.B., Sanders, S.J., Stevens, C., Sabo, A., McGrath, L.M., Kosmicki, J.A., Rehnström, K., Mallick, S., Kirby, A., et al. (2014). A framework for the interpretation of de novo mutation in human disease. Nat. Genet. *46*, 944–950.
18. Zaru, R., Orchard, S.; and UniProt Consortium (2023). UniProt Tools: BLAST, Align, Peptide Search, and ID Mapping. Curr. Protoc. *3*, e697.
19. Robins, D.L., Casagrande, K., Barton, M., Chen, C.M.A., Dumont-Mathieu, T., and Fein, D. (2014). Validation of the modified checklist for autism in toddlers, revised with follow-up (M-CHAT-R/F). Pediatrics *133*, 37–45.
20. Auyeung, B., Simon, A.E., Ae, B.-C., Ae, S.W., and Allison, C. (2008). The Autism Spectrum Quotient: Children's Version

(AQ-Child). J. Autism Dev. Disord. *38*, 1230–1240. https://doi.org/10.1007/s10803-007-0504-z.
21. Baron-Cohen, S., Hoekstra, R.A., Knickmeyer, R., and Wheelwright, S. (2006). The Autism-Spectrum Quotient (AQ) - Adolescent version. J. Autism Dev. Disord. *36*, 343–350.
22. Pennington, L., Virella, D., Mjøen, T., da Graça Andrada, M., Murray, J., Colver, A., Himmelmann, K., Rackauskaite, G., Greitane, A., Prasauskiene, A., et al. (2013). Development of The Viking Speech Scale to classify the speech of children with cerebral palsy. Res. Dev. Disabil. *34*, 3202–3210.
23. Caynes, K., Rose, T.A., Theodoros, D., Burmester, D., Ware, R. S., and Johnston, L.M. (2019). The Functional Communication Classification System: extended reliability and concurrent validity for children with cerebral palsy aged 5 to 18 years. Dev. Med. Child Neurol. *61*, 805–812.
24. Sadeh, A. (2004). A brief screening questionnaire for infant sleep problems: validation and findings for an Internet sample. Pediatrics *113*, e570–e577.
25. Chervin, R.D., Hedger, K., Dillon, J.E., and Pituch, K.J. (2000). Pediatric sleep questionnaire (PSQ): validity and reliability of scales for sleep-disordered breathing, snoring, sleepiness, and behavioral problems. Sleep Med. *1*, 21–32.
26. Sellers, D., Mandy, A., Pennington, L., Hankins, M., and Morris, C. (2014). Development and reliability of a system to classify the eating and drinking ability of people with cerebral palsy. Dev. Med. Child Neurol. *56*, 245–251.
27. Eliasson, A.C., Ullenhag, A., Wahlström, U., and Krumlinde-Sundholm, L. (2017). Mini-MACS: development of the Manual Ability Classification System for children younger than 4 years of age with signs of cerebral palsy. Dev. Med. Child Neurol. *59*, 72–78.
28. Eliasson, A.C., Krumlinde-Sundholm, L., Rösblad, B., Beckung, E., Arner, M., Ohrvall, A.M., and Rosenbaum, P. (2006). The Manual Ability Classification System (MACS) for children with cerebral palsy: scale development and evidence of validity and reliability. Dev. Med. Child Neurol. *48*, 549–554.
29. Palisano, R., Rosenbaum, P., Walter, S., Russell, D., Wood, E., and Galuppi, B. (1997). Development and reliability of a system to classify gross motor function in children with cerebral palsy. Dev. Med. Child Neurol. *39*, 214–223.
30. Baranello, G., Signorini, S., Tinelli, F., Guzzetta, A., Pagliano, E., Rossi, A., Foscan, M., Tramacere, I., Romeo, D.M.M., Ricci, D.; and VFCS Study Group (2020). Visual Function Classification System for children with cerebral palsy: development and validation. Dev. Med. Child Neurol. *62*, 104–110.
31. Harrison, P.L., and Oakland, T. (2018). Adaptive Behavior Assessment System. In Encyclopedia of Clinical Neuropsychology, Third Edition (Western Psychological Services), pp. 57–60. https://doi.org/10.1007/978-3-319-57111-9_1506.
32. Achenbach, T.M. (2017). Achenbach System of Empirically Based Assessment (ASEBA). In Encyclopedia of Clinical Neuropsychology, K. Jeffrey, J. DeLuca, and B. C, eds. (Springer International Publishing), pp. 1–7. https://doi.org/10.1007/978-3-319-56782-2_1529-3.
33. R Core Team (2021). *R: A Language and Environment for Statistical Computing*. R Foundation for Statistical Computing. URL https://www.R-project.org/.
34. Bürkner, P.C. (2017). brms: An R Package for Bayesian Multilevel Models Using Stan. J Stat Softw *80*, 1–28.
35. Therneau, T. (2023). A Package for Survival Analysis in R. https://CRAN.R-project.org/package=survival.
36. Morales, J., Pujar, S., Loveland, J.E., Astashyn, A., Bennett, R., Berry, A., Cox, E., Davidson, C., Ermolaeva, O., Farrell, C.M., et al. (2022). A joint NCBI and EMBL-EBI transcript set for clinical genomics and research. Nature *604*, 310–315.
37. Leung, S.K., Jeffries, A.R., Castanho, I., Jordan, B.T., Moore, K., Davies, J.P., Dempster, E.L., Bray, N.J., O'Neill, P., Tseng, E., et al. (2021). Full-length transcript sequencing of human and mouse cerebral cortex identifies widespread isoform diversity and alternative splicing. Cell Rep. *37*, 110022.
38. Chan, J.J., Zhang, B., Chew, X.H., Salhi, A., Kwok, Z.H., Lim, C.Y., Desi, N., Subramaniam, N., Siemens, A., Kinanti, T., et al. (2022). Pan-cancer pervasive upregulation of 3′ UTR splicing drives tumourigenesis. Nat. Cell Biol. *24*, 928–939.
39. Cheng, J., Novati, G., Pan, J., Bycroft, C., Žemgulytė, A., Applebaum, T., Pritzel, A., Wong, L.H., Zielinski, M., Sargeant, T., et al. (2023). Accurate proteome-wide missense variant effect prediction with AlphaMissense. Science *381*, eadg7492.
40. Fu, J.M., Satterstrom, F.K., Peng, M., Brand, H., Collins, R.L., Dong, S., Wamsley, B., Klei, L., Wang, L., Hao, S.P., et al. (2022). Rare coding variation provides insight into the genetic architecture and phenotypic context of autism. Nat. Genet. *54*, 1320–1331.
41. Kaplanis, J., Samocha, K.E., Wiel, L., Zhang, Z., Arvai, K.J., Eberhardt, R.Y., Gallone, G., Lelieveld, S.H., Martin, H.C., McRae, J.F., et al. (2020). Evidence for 28 genetic disorders discovered by combining healthcare and research data. Nature *586*, 757–762.
42. Fischbach, G.D., and Lord, C. (2010). The Simons Simplex Collection: a resource for identification of autism genetic risk factors. Neuron *68*, 192–195.
43. Lynch, M. (2010). Rate, molecular spectrum, and consequences of human mutation. Proc. Natl. Acad. Sci. USA *107*, 961–968.
44. Jaganathan, K., Kyriazopoulou Panagiotopoulou, S., McRae, J.F., Darbandi, S.F., Knowles, D., Li, Y.I., Kosmicki, J.A., Arbelaez, J., Cui, W., Schwartz, G.B., et al. (2019). Predicting Splicing from Primary Sequence with Deep Learning. Cell *176*, 535–548.e24.
45. WHO Child Growth Standards. https://www.who.int/tools/child-growth-standards/standards/weight-for-age.
46. Miroševič, Š., Khandelwal, S., Sušjan, P., Žakelj, N., Gosar, D., Forstnerič, V., Lainščček, D., Jerala, R., and Osredkar, D. (2022). Correlation between Phenotype and Genotype in CTNNB1 Syndrome: A Systematic Review of the Literature. Int. J. Mol. Sci. *23*, 12564.
47. Hadjihannas, M.V., Brückner, M., Jerchow, B., Birchmeier, W., Dietmaier, W., and Behrens, J. (2006). Aberrant Wnt-Catenin Signaling Can Induce Chromosomal Instability in Colon Cancer. Proc. Natl. Acad. Sci. USA *103*, 10747–10752. https://doi.org/10.1073/pnas.0604206103.
48. Garone, G., Innocenti, A., Grasso, M., Mandarino, A., Capuano, A., Della Bella, G., Frascarelli, F., Diodato, D., Onesimo, R., Zampino, G., et al. (2024). Movement disorder phenotype in CTNNB1-syndrome: A complex but recognizable phenomenology. Parkinsonism Relat. Disord. *126*, 107057.
49. Servais, L., Camino, E., Clement, A., McDonald, C.M., Lukawy, J., Lowes, L.P., Eggenspieler, D., Cerreta, F., and Strijbos, P. (2021). First Regulatory Qualification of a Novel Digital Endpoint in Duchenne Muscular Dystrophy: A Multi-Stakeholder Perspective on the Impact for Patients and for Drug Development in Neuromuscular Diseases. Digit. Biomark. *5*, 183–190.

HGGA, Volume 6

Supplemental information

Genotypic, functional, and phenotypic characterization in CTNNB1 neurodevelopmental syndrome

Nina Žakelj, David Gosar, Špela Miroševič, Stephan J. Sanders, Alicia Ljungdahl, Sayeh Kohani, Shouhe Huang, Lok I Leong, Ying An, Miou-Jing Teo, Fiona Moultrie, Roman Jerala, Duško Lainšček, Vida Forstnerič, Petra Sušjan, Leszek Lisowski, Andrea Perez-Iturralde, Jasna Oražem Mrak, Ho Yin Edwin Chan, and Damjan Osredkar

Supplementary Text

VSS: "The four levels include: (1) speech is not affected by motor disorder; (2) speech is imprecise but usually understandable to unfamiliar listeners; (3) speech is unclear and not usually understandable to unfamiliar listeners out of context; (4) no comprehensible speech. "

FCSS: "The five levels include: (1) effective communicator in most situations; (2) effective communicator in most situations, but does need some help; (3) an effective communicator in most situations and can communicate small ranges of messages and topics to most familiar people; (4) assistance is required in most situations, especially with unfamiliar people and environments; (5) communicates unintentionally using movements and behaviour."

GMFCS: "The five levels include: (1) Children walk at home, school, outdoors and in the community. They can climb stairs without the use of a railing. Children perform gross motor skills such as running and jumping, but speed, balance and coordination are limited; (2) Children walk in most settings and climb stairs holding onto a railing. They may experience difficulty walking long distances and balancing on uneven terrain, inclines, in crowded areas or confined spaces. Children may walk with physical assistance, a handheld mobility device or used wheeled mobility over long distances. Children have only minimal ability to perform gross motor skills such as running and jumping; (3) Children walk using a hand-held mobility device in most indoor settings. They may climb stairs holding onto a railing with supervision or assistance. Children use wheeled mobility when travelling long distances and may self-propel for shorter distances; (4) Children use methods of mobility that require physical assistance or powered mobility in most settings. They may walk for short distances at home with physical assistance or use powered mobility or a body support walker when positioned. At school, outdoors and in the community children are transported in a manual wheelchair or use powered mobility; (5) Children are transported in a manual wheelchair in all settings. Children are limited in their ability to maintain antigravity head and trunk postures and control leg and arm movements."

VFCS: (1) Uses visual function easily and successfully in vision-related activities; (2) Uses visual function successfully but needs self-initiated compensatory strategies; (3) Uses visual function but needs some adaptations; (4) Uses visual function in very adapted environments but performs just part of vision-related activities; (5) Does not use visual function even in very adapted environments.

EADCS: (1) Eats and drinks safely and efficiently; (2) Eats and drinks safely but with some limitations to efficiency; (3) Eats and drinks with some limitations to safety. There maybe limitations to efficiency; (4) Eats and drinks with significant limitations to safety; (5) Unable to eat or drink safely - tube feeding may be considered to provide nutrition.

Supplementary Figures

Figure S1. Alternative splicing transcripts for the *CTNNB1*. Genomic coordinates GRCh38/hg38 for a 49k nucleotide region of chromosome 3 are shown at the top. There is a ~13k gap to help highlight regions of interest. Four features of note are named in red with light red bars showing the genomic region across the figure. Nucleotide-resolution of the median read counts of RNA-seq gene expression data from 176 *postmortem* human frontal cortices (PMID: 32268104) are shown organized by developmental stage; data are auto-scaled. The protein-coding (CDS) exon number is show for comparability to Fig. 2. All GENCODEv46 transcripts for the *CTNNB1* gene are represented, including the MANE Select transcript ENST00000349496.11, NM_001904.4 at the top in light blue. Protein-coding transcripts are in dark blue, while non-coding transcripts are shown in grey. Epigenetic data from ENCODE, specifically the H3K27ac marker of actively expressed regions across cell types, is represented under the gene transcripts. Species conservation across 100 vertebrates is represented at the bottom, including PhyloP scores (top) and homology across species (bottom). We note that the TSS region is shown as absent in *Rhesus*, however, BLAT of the human TSS DNA sequence correctly identifies the corresponding TSS region in *Rhesus*, suggesting a cross-species genome alignment error in this representation. Image was generated from UCSC genome browser (PMID: 36420891).

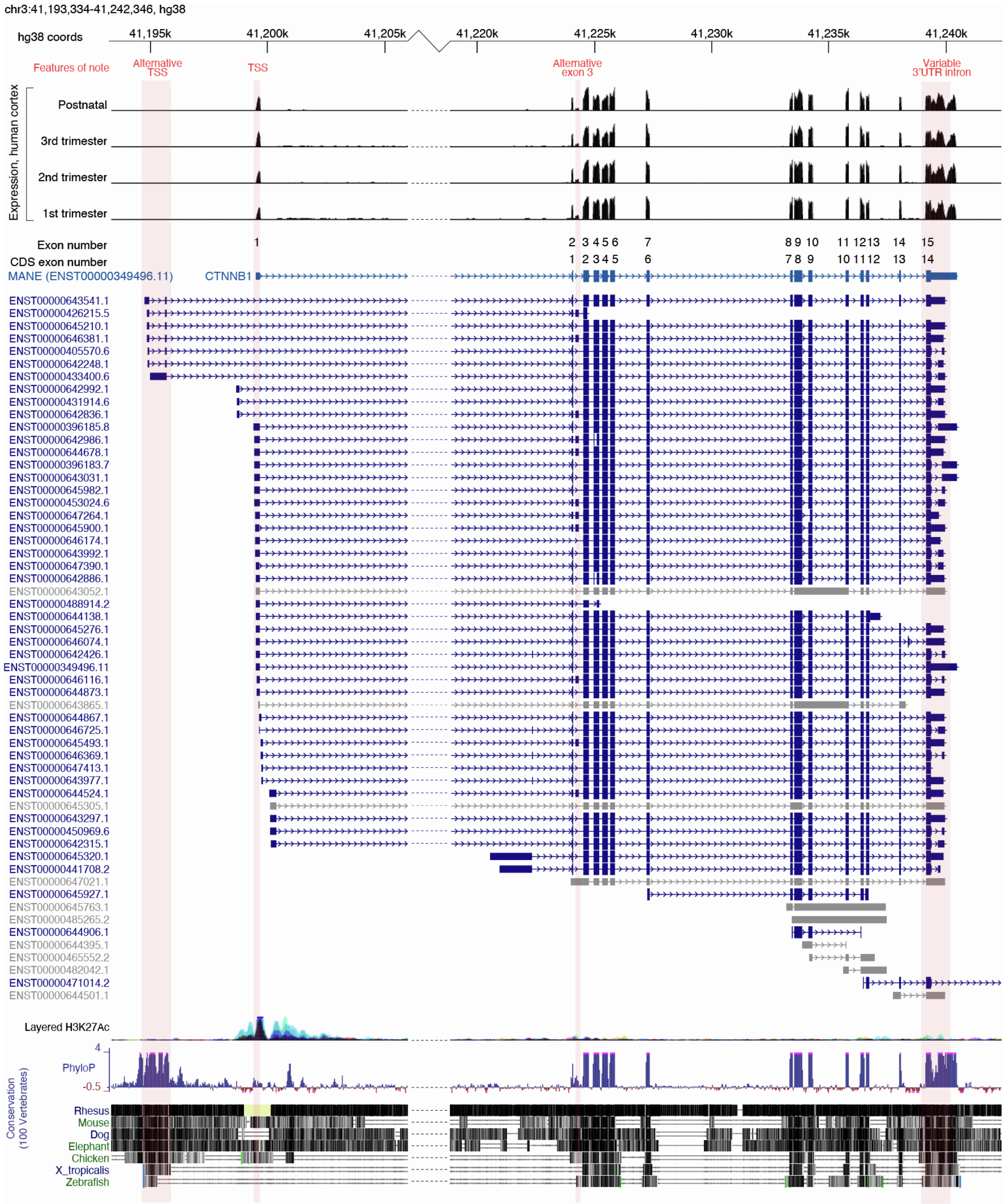
chr3:41,193,334-41,242,346, hg38
hg38 coords
41,195k
41,200k
41,205k
41,220k
41,225k
41,230k
41,235k
41,240k
Features of note
Alternative TSS
TSS
Alternative exon 3
Variable 3'UTR intron
Expression, human cortex
Postnatal
3rd trimester
2nd trimester
1st trimester
Exon number
1
2 3 4 5 6
7
8 9 10
11 12 13
14
15
CDS exon number
1 2 3 4 5
6
7 8 9
10 11 12
13
14
MANE (ENST00000349496.11)
CTNNB1
ENST00000643541.1
ENST00000426215.5
ENST00000645210.1
ENST00000646381.1
ENST00000405570.6
ENST00000642248.1
ENST00000433400.6
ENST00000642992.1
ENST00000431914.6
ENST00000642836.1
ENST00000396185.8
ENST00000642986.1
ENST00000644678.1
ENST00000396183.7
ENST00000643031.1
ENST00000645982.1
ENST00000453024.6
ENST00000647264.1
ENST00000645900.1
ENST00000646174.1
ENST00000643992.1
ENST00000647390.1
ENST00000642886.1
ENST00000643052.1
ENST00000488914.2
ENST00000644138.1
ENST00000645276.1
ENST00000646074.1
ENST00000642426.1
ENST00000349496.11
ENST00000646116.1
ENST00000644873.1
ENST00000643865.1
ENST00000644867.1
ENST00000646725.1
ENST00000645493.1
ENST00000646369.1
ENST00000647413.1
ENST00000643977.1
ENST00000644524.1
ENST00000645305.1
ENST00000643297.1
ENST00000450969.6
ENST00000642315.1
ENST00000645320.1
ENST00000441708.2
ENST00000647021.1
ENST00000645927.1
ENST00000645763.1
ENST00000485265.2
ENST00000644906.1
ENST00000644395.1
ENST00000465552.2
ENST00000482042.1
ENST00000471014.2
ENST00000644501.1
Layered H3K27Ac
4
PhyloP
-0.5
Conservation (100 Vertebrates)
Rhesus
Mouse
Dog
Elephant
Chicken
X_tropicalis
Zebrafish

Figure S2. Developmental patterns of exon expression for the *CTNNB1*. A) The expression of 128 exon subdivisions, as defined by GENCODE transcripts and DEX-seq (Table SX), are represented across development. The lines represent the Lowess smoothed log-scaled counts per million (CPM) values across development from 176 *postmortem* human frontal cortices (PMID: 32268104). **B)** The correlation between the ratio of CPM expression between pairs of protein-coding exon subdivisions (log-scaled) and developmental stage (post-conceptual days, log-scaled) for *CTNNB1* was assessed with a linear model. The R^2 value of each exon pair is shown as a heat map with 'hot' colors representing exon pairs with high R^2 values for which variation in the ratio is correlated with developmental age, i.e., pairs of exons that show substantially different expression across development (PMID: 34425903).

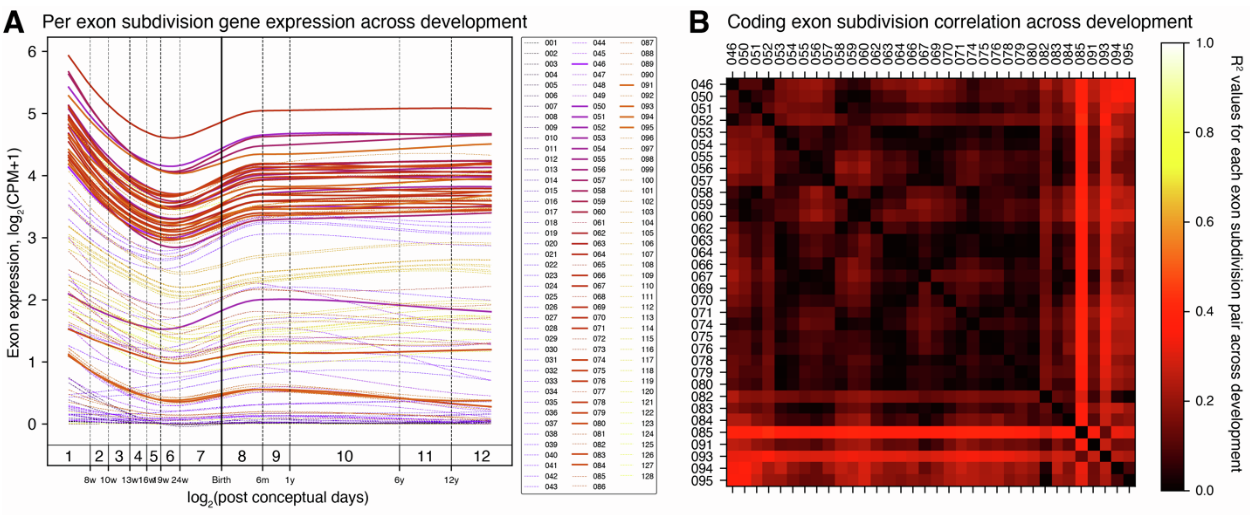

Figure S3. Number of participants in clinical trial per country and the world-map of countries with involved participants.

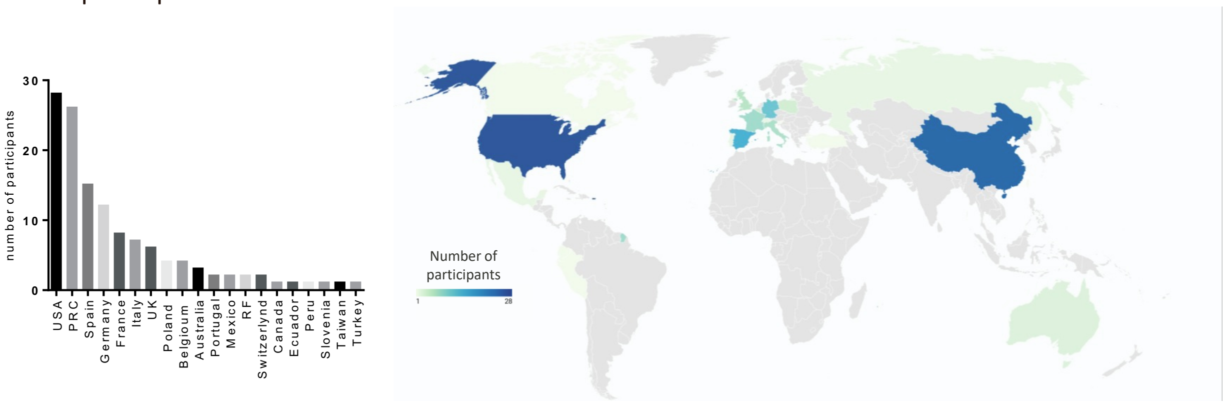

Figure S4. β-catenin protein expression in various CTNNB1 mutant constructs. WT – wildtype.

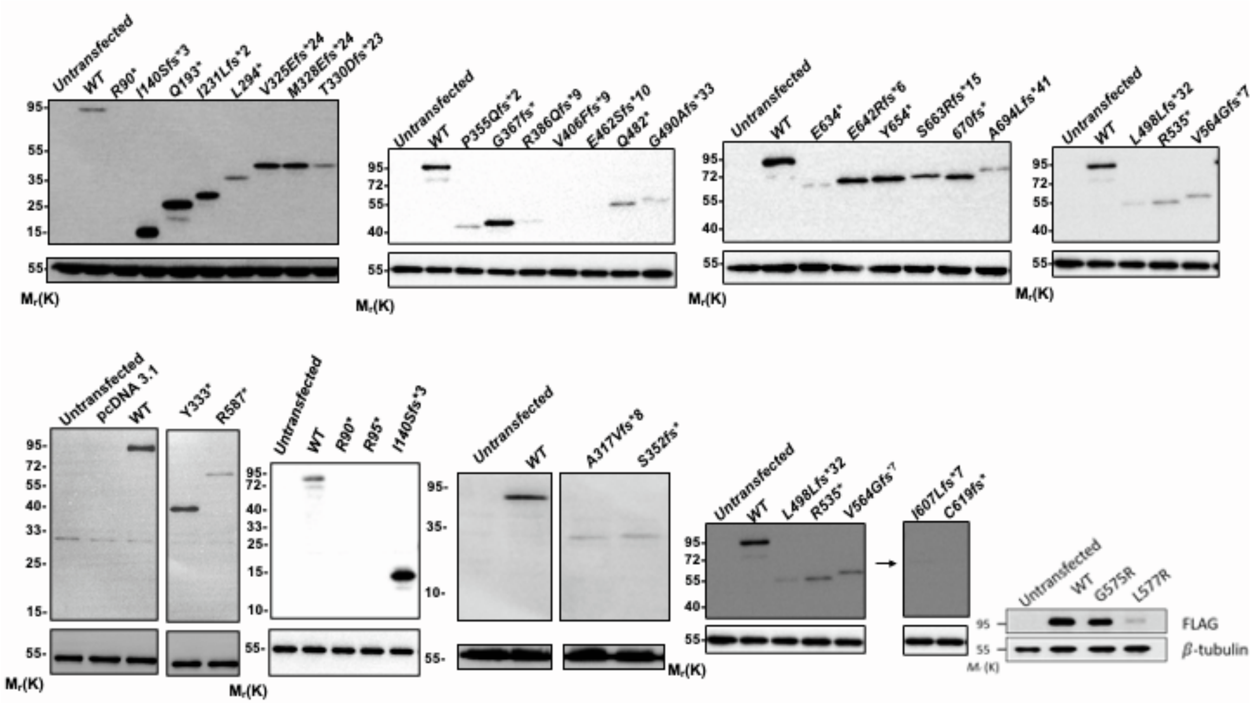

Figure S5. Thermal stability of β-catenin mutants determined by cellular thermal shift assay (CETSA). A) Cellular proteins extracted from SK-N-MC cells expressing *CTNNB1*^{WT}, *CTNNB1*^{G575R}, *or CTNNB1*^{L577R} were exposed to temperatures ranging from 40°C to 68°C and the soluble fractions were collected for western blot analysis. **B)** The relative band intensity of FLAG signal was quantified and fitted using the Boltzmann sigmoidal equation. Results are from three independent experiments. Error bars denote standard error of the mean (SEM). *Tm, melting temperature; ICE, ice control; RT, room temperature.*

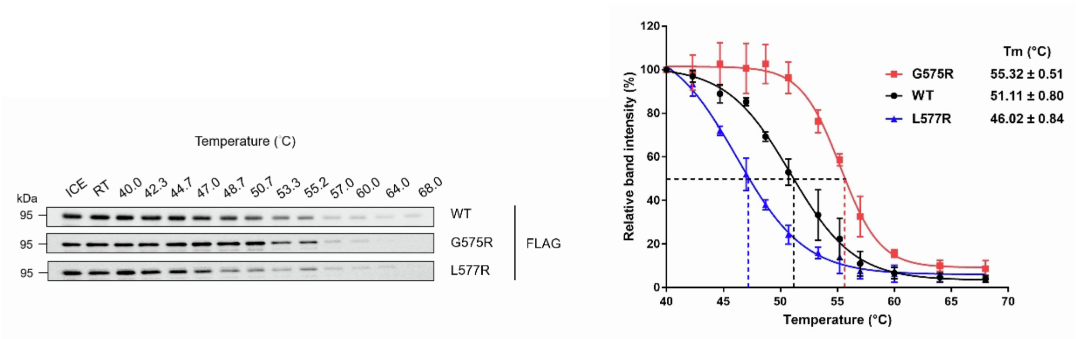

Figure S6. Prenatal and perinatal characteristics. Percentage of (A) prenatal and (B) perinatal risk factors in CTNNB1 syndrome participants with 95% confidence intervals.

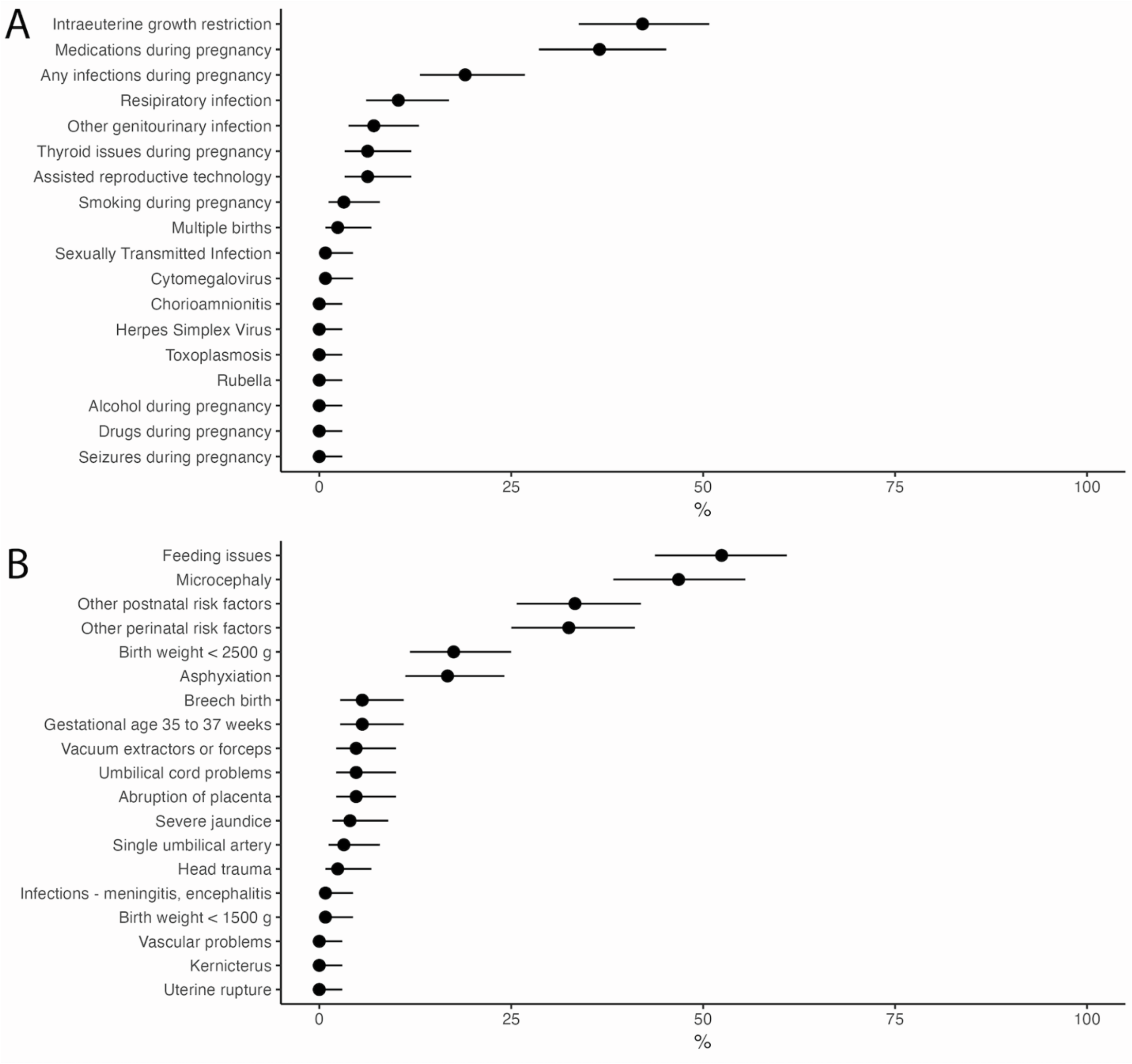

Figure S7. Ophthalmologic signs/symptoms in CTNNB1 syndrome patients as reported by parents with 95% confidence intervals.

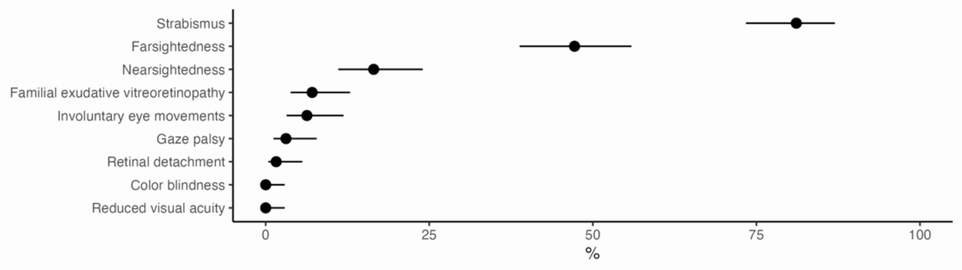

Figure S8. ABAS-3 scores for adaptive skills subdomains (A) and adaptive skills domains (B) for CTNNB1 syndrome patients (N = 114).

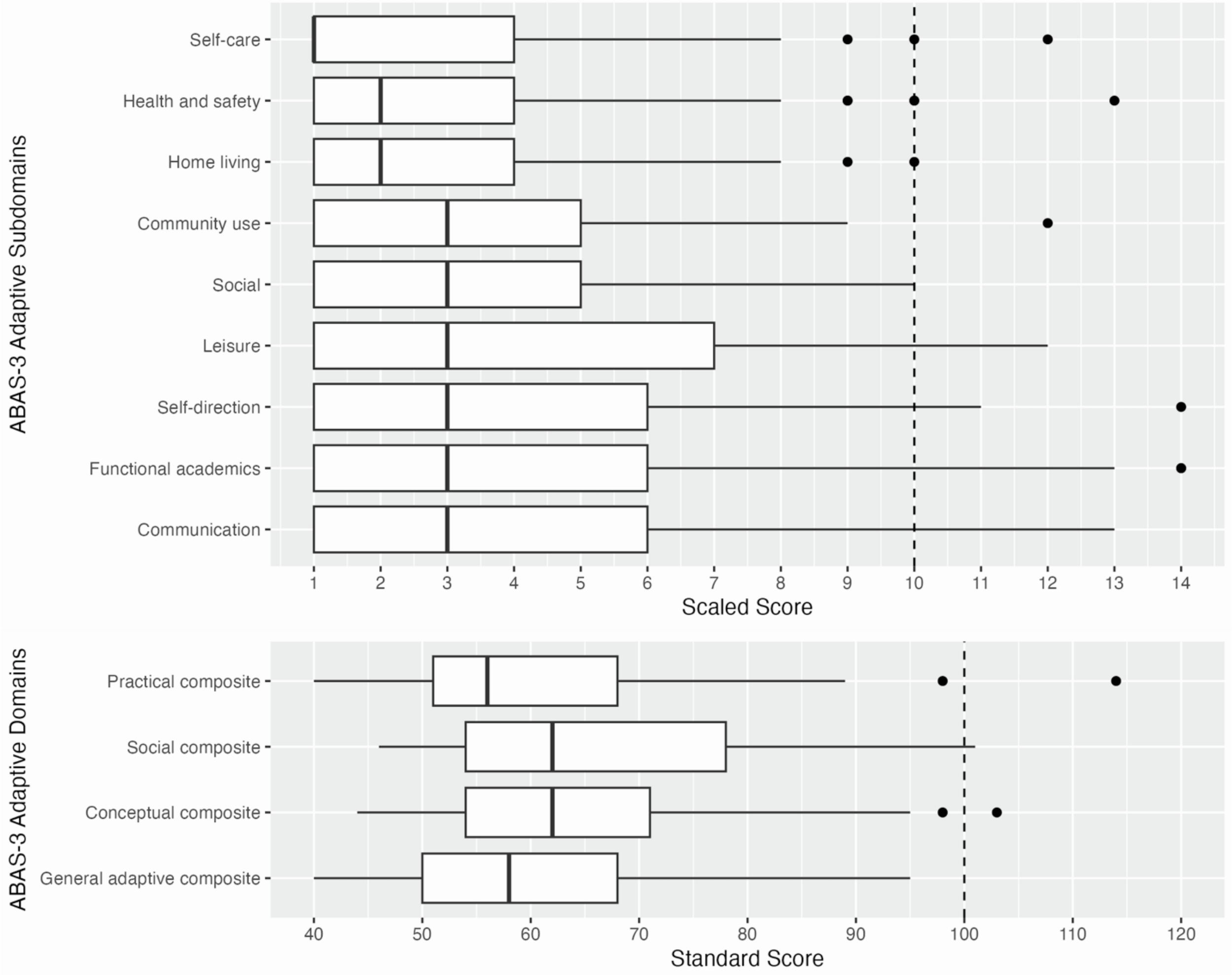

Supplementary Tables

Table S1. All SNVs (separate file)
Supplementary Table 1.xlsx

Table S2. Variant Mapper and Clinical Data (separate file)
Supplementary Table 2.xlsx

Table S3. Exon subdivisions (separate file)
Supplementary Table 3.xlsx

Table S4. Estimated protein size of overexpressed mutant β-catenin proteins.

Mutant protein (overexpressed)	Observed protein size (kDa)
R90*	Not detected
R95*	Not detected
I140Sfs*3	~15
Q193*	~25
I231Lfs*2	~27
L294*	~35
V325Efs*24	~38
M328Efs*24	~38
T330Dfs*23	~38
Y333*	~38
P355Qfs*2	~40
G367fs*	~42
R386Qfs*9	~42
V406Ffs*9	Not detected
E462Sfs*10	Not detected
Q482*,	~52
G490Afs*33	~54
L498Lfs*32,	~54
R535*,	~54
V564Gfs*7	~56
R587*	~64
I607Lfs*7	~67
C619fs*	Not detected
E634*	~69
E642Rfs*6	~70
Y654*	~70
S663Rfs*15	~71
A694Lfs*41	~72
Y670fs*	~70

Table S5. Standardized scores on the ABAS-3 adaptive skill domains and the General Adaptive Composite (GAC) of patients with different types of *CTNNB1* variants. *Me* – median of posterior distribution*; HDI* $_{low}$ – lower bound of the 89% highest density interval for the posterior distribution;*HDI* $_{high}$ – upper bound of the 95% highest density interval for the posterior distribution; *p*d – probability of direction (likelihood of an effects existence); ROPE – proportion outside of Region of Practical Equivalence for neurotypical individuals (ROPE).

	Me	*HDI* $_{low}$	*HDI* $_{high}$	*pd*	*ROPE*
**General Adaptive Composite**					
Missense variant	68.6	46.8	88.3	<.001	<.001
Nonsense variant	52.4	36.8	62.6	<.001	.001
Frameshift variant	47.6	31.5	58.6	<.001	.001
Whole gene deletion	63.8	39.2	86.8	.400	.004
Splice variant	55.4	35.7	72.0	.100	.003
**Conceptual Domain**					
Missense variant	84.6	69.0	97.9	<.001	<.001
Nonsense variant	63.4	59.9	67.0	<.001	.001
Frameshift variant	64.1	60.7	67.4	<.001	<.001
Whole gene deletion	60.5	41.0	73.3	<.001	.001
Splice variant	62.2	50.4	70.5	<.001	<.001
**Social Domain**					
Missense variant	83.0	67.0	95.7	<.001	<.001
Nonsense variant	66.8	63.4	70.3	<.001	.001
Frameshift variant	64.6	60.4	68.2	<.001	.001
Whole gene deletion	67.4	51.1	79.2	.100	.002
Splice variant	63.9	52.3	73.6	<.001	.001
**Practical Domain**					
Missense variant	63.5	39.9	85.3	<.001	<.001
Nonsense variant	50.8	34.1	62.9	.100	.004
Frameshift variant	40.8	23.5	56.0	<.001	.001
Whole gene deletion	73.7	45.9	99.4	.300	.003
Splice variant	57.9	36.8	76.0	.300	.005

Table S6. Censored regression results for predicting age at indepedent walking based on functional status of variant (LoF dominant negative vs. LoF not dominant negative).

	LoF DN			LoF nDN				
		95% HDI			95% HDI			
	Est.	lw. bound	up. bound	Est.	lw. bound	up. bound	*BF*	*pd*
Age at independent walking (in months)	36.0	23.7	51.7	33.7	27.7	40.4	1.65	0.622

Note: LoF DN - LoF dominant negative, LoF nDN - LoF not dominant negative

Table S7. Regression results for predicting adaptive skills based on functional status of variant (LoF dominant negative vs. LoF not dominant negative).

	LoF DN			LoF nDN				
		95% HDI			95% HDI			
	Est.	lw. bound	up. bound	Est.	lw. bound	up. bound	*BF*	*pd*
ABAS-3 Scales								
GAC	54.1	38.1	66.8	53.7	40.5	63.0	0.81	0.449
Conceptual Composite	61.2	49.2	70.7	62.8	54.1	68.9	1.63	0.619
Social Composite	64.5	51.2	74.6	63.1	50.3	69.8	0.53	0.347
Practical Composite	49.2	24.1	66.0	53.4	36.3	64.3	2.10	0.678

Note: LoF DN - LoF dominant negative, LoF nDN - LoF not dominant negative

Table S8. Regression results for predicting symptoms of psychopathology based on functional status of variant (LoF dominant negative vs. LoF not dominant negative).

	LoF DN			LoF nDN				
		95% HDI			95% HDI			
	Est.	lw. bound	up. bound	Est.	lw. bound	up. bound	*BF*	*pd*
ASEBA Scales								
Anxious/Depressed	57.9	53.5	61.9	60.1	57.8	62.7	6.83	0.872
Withdrawn Behavior	61.8	55.9	67.4	65.7	62.6	68.8	8.10	0.890
Somatic Complaints	58.0	54.4	61.5	58.2	56.1	60.5	1.19	0.543
Thought Problems	75.3	64.6	85.2	72.9	67.8	78.2	0.52	0.342
Autism Spectrum	62.2	56.6	68.1	66.7	63.5	69.8	9.46	0.904
Attention Problems	66.2	60.8	71.7	69.5	66.6	72.5	6.25	0.862
Aggressive Behavior	60.4	55.7	65.1	62.0	59.2	65.1	3.05	0.753

Note: LoF DN - LoF dominant negative, LoF nDN - LoF not dominant negative

Table S9. Ordinal regression results for predicting the functional assessment scale scores of patientswith a LoF dominant negative variant compared to patients with a dominant negative variant.

		95% HDI			
	Est.	lw. bound	up. bound	*BF*	*pd*
Functional Scales					
VSS	-0.66	-2.55	1.36	3.1	0.754
FCCS	-1.01	-2.76	0.77	6.9	0.873
GMFCS	-0.44	-1.65	0.83	3.1	0.756
VFCS	-0.2	-1.54	1.12	1.6	0.621
EDACS	-0.15	-1.8	1.49	1.3	0.571

Note: GMFCS – Gross Motor Function Classification System; EADCS – Eating and Drinking Ability Classification System; FCCS – Functional Communication Classification System; VFCS – Visual Function Classification System; VSS – Viking speech scale

Table S10. Censored regression results for the age at independent walking by patients with a LoF dominant negative variant compared to patients with a dominant negative, LoF or LoF presumed variant.

	LoF DN			LoF nDN+				
		95% HDI			95% HDI			
	Est.	lw. bound	up. bound	Est.	lw. bound	up. bound	*BF*	*pd*
Age at independent walking (in months)	36.0	23.7	51.7	39.3	35.1	43.6	0.48	0.323

Note: LoF DN - LoF dominant negative, LoF nDN+ - LoF not dominant negative, LoF, LoF presumed.

Table S11. Regression results for predicting the ABAS-3 adaptive skills of patients with a LoF dominant negative variant compared to patients with a dominant negative, LoF or LoF presumed variant.

	LoF DN			LoF nDN+				
		95% HDI			95% HDI			
	Est.	lw. bound	up. bound	Est.	lw. bound	up. bound	*BF*	*pd*
ABAS-3 Scales								
GAC	51.3	31.8	66.3	53.5	39.5	60.9	1.35	0.575
Conceptual Composite	61.7	51.0	68.9	63.8	61.3	66.5	2.93	0.746
Social Composite	65.6	58.0	72.1	65.5	63.0	68.2	0.96	0.489
Practical Composite	50	26.3	65.8	54.8	43.2	61.6	2.21	0.689

Note: LoF DN - LoF dominant negative, LoF nDN+ - LoF not dominant negative, LoF, LoF presumed.

Table S12. Regression results for predicting symptoms of psychopathology in patients with a LoF dominant negative variant compared to patients with a dominant negative, LoF or LoF presumed variant.

	LoF DN			LoF nDN+				
		95% HDI			95% HDI			
	Est.	lw. bound	up. bound	Est.	lw. bound	up. bound	*BF*	*pd*
ASEBA Scales								
Anxious/Depressed	58.6	54.3	61.8	60.5	59.1	62.1	8.72	0.897
Withdrawn Behavior	62.5	56.7	68.3	65.6	63.6	67.5	5.63	0.849
Somatic Complaints	58.3	55.0	61.0	58.8	57.5	60.3	1.77	0.639
Thought Problems	73.9	63.8	84.4	73.2	70.3	76.0	0.82	0.451
Autism Spectrum	62.1	55.0	68.8	69.1	66.7	71.5	26.93	0.964
Attention Problems	66.6	60.6	72.0	69.4	67.5	71.3	4.65	0.823
Aggressive Behavior	61.0	56.2	65.0	63.1	61.3	65.1	6.43	0.865

Note: LoF DN - LoF dominant negative, LoF nDN+ - LoF not dominant negative, LoF, LoF presumed.

Table S13. Ordinal regression results for predicting the functional assessment scale scores of patients with a LoF dominant negative variant compared to patients with a dominant negative variant, LoF or LoF presumed variant.

		95% HDI			
	Est.	lw. bound	up. bound	*BF*	*pd*
Functional Scales					
VSS	-0.46	-2.34	1.42	2.2	0.686
FCCS	-0.81	-2.52	0.81	5.1	0.837
GMFCS	0.05	-1.08	1.22	0.9	0.534
VFCS	-0.08	-1.29	1.13	1.2	0.551
EDACS	-0.41	-1.91	1.17	2.4	0.703

Note: GMFCS – Gross Motor Function Classification System; EADCS – Eating and Drinking Ability Classification System; FCCS – Functional Communication Classification System; VFCS – Visual Function Classification System; VSS – Viking speech scale

Table S14. Censored regression results for predicting age at indepedent walking based on variant type (age in months).

	Est.	95% HDI lw. bound	95% HDI up. bound	*BF*	*pd*
Missense variant	27.7	18.4	42.5		
Nonsense variant	41.0	35.2	47.0	15.21	0.938
Frameshift	39.2	33.3	45.2	11.00	0.917
Whole gene deletion	45.7	27.1	79.7	16.21	0.942
Splice	35.7	25.0	50.9	4.60	0.821

Table S15. Regression results for predicting adaptive skills based on variant type.

	Est.	95% HDI lw. bound	95% HDI up. bound	*BF*	*pd*
GAC					
Missense variant	76.9	51.8	100.8		
Nonsense variant	52.9	36.1	63.5	89.91	0.989
Frameshift	46.7	29.4	58.4	391.16	0.997
Whole gene deletion	61.5	31.5	88.7	3.77	0.79
Splice	53.9	32.5	71.6	21.25	0.955
Conceptual Composite					
Missense variant	90.2	75.1	101		
Nonsense variant	63.3	59.8	66.8	665.67	0.999
Frameshift	63.8	60.4	67.1	464.12	0.998
Whole gene deletion	58.5	31.5	72.2	407.16	0.998
Splice	61.5	48.2	70.3	376.36	0.997
Social Composite					
Missense variant	87	72	98.6		
Nonsense variant	66.8	63.4	70.5	79.97	0.988
Frameshift	64.1	59.5	67.9	185.92	0.995
Whole gene deletion	66.5	46.9	79.5	39.73	0.975
Splice	63.5	51.1	73.4	111.36	0.991
Practical Composite					
Missense variant	71.9	42.1	98.3		

Nonsense variant	51.3	34	64.1	18.23	0.948
Frameshift	40.5	22.1	55.9	121.7	0.992
Whole gene deletion	72.1	38.3	102.9	0.95	0.487
Splice	56.8	33.7	75.6	4.32	0.812

Table S16. Regression results for predicting symptoms of psychopathology based on variant type.

		95% HDI			
	Est.	lw. bound	up. bound	*BF*	*pd*
Anxious/Depressed					
Missense variant	56.6	47.5	62.3		
Nonsense variant	59.8	58	61.8	6.17	0.861
Frameshift	60.9	59.1	62.8	16.11	0.942
Whole gene deletion	56.2	43.1	64.3	0.96	0.489
Splice	62.4	57.3	67.1	16.59	0.943
Withdrawn Behavi					
Missense variant	67.7	56.5	77.2		
Nonsense variant	64.4	61.8	67.2	0.41	0.29
Frameshift	65.5	62.9	68.1	0.55	0.354
Whole gene deletion	62.5	48.6	74.5	0.36	0.265
Splice	66.9	58.9	74.9	0.87	0.467
Somatic Complaints					
Missense variant	64.0	55.9	69.8		
Nonsense variant	58.6	57.0	60.3	0.12	0.111
Frameshift	58.7	57.2	60.4	0.13	0.117
Whole gene deletion	54.6	42.6	61.7	0.05	0.052
Splice	61.8	56.6	65.6	0.43	0.298
Thought Problems					
Missense variant	73.4	57.8	88.9		
Nonsense variant	72.1	68.7	75.8	0.78	0.439
Frameshift	76.2	72.2	80.3	1.69	0.628
Whole gene deletion	51.7	35.4	68.2	0.03	0.029
Splice	72.5	62.7	82.0	0.86	0.462
Autism Spectrum					
Missense variant	67.5	55.6	80.4		

Nonsense variant	66.3	62.6	69.9	0.73	0.422
Frameshift	68.6	65.6	71.6	1.27	0.56
Whole gene deletion	67.5	51.2	87.1	1.00	0.501
Splice	79.2	68.7	89.2	11.11	0.917
Attention Problems					
Missense variant	67.6	57.1	77.8		
Nonsense variant	68.5	65.7	71.3	1.29	0.564
Frameshift	69.5	66.8	72.1	1.73	0.634
Whole gene deletion	63.6	49.7	75.6	0.45	0.312
Splice	71.8	64.2	79.1	2.89	0.743
Aggressive Behavior					
Missense variant	60.9	50.9	68.6		
Nonsense variant	62.2	59.8	64.6	1.68	0.627
Frameshift	63.3	61.1	65.8	2.79	0.736
Whole gene deletion	58.0	43.9	66.7	0.48	0.326
Splice	67.7	60.3	73.8	10.37	0.912

Table S17. Ordinal regression results for predicting scores on functional assessment scales based on variant type (missense variants vs. nonsense variants, frameshift variants, whole gene deletions and splice variants).

		95% HDI			
	Est.	lw. bound	up. bound	*BF*	*pd*
VSS					
Nonsense variant	1.94	-0.69	4.74	12.6	0.927
Frameshift	2.36	-0.2	5.20	25.7	0.963
Whole gene deletion	3.44	-0.28	7.54	29.5	0.967
Splice	1.42	-1.47	4.52	4.9	0.831
FCCS					
Nonsense variant	1.50	-0.96	3.96	8.5	0.895
Frameshift	1.24	-1.24	3.62	5.6	0.849
Whole gene deletion	5.00	1.01	9.01	195.1	0.995
Splice	0.77	-2.1	3.61	2.4	0.706
GMFCS					
Nonsense variant	5.35	1.50	10.94	>1000.00	>.999

Frameshift	5.89	2.10	11.59	>1000.00	>.999
Whole gene deletion	6.59	2.30	12.69	>1000.00	>.999
Splice	6.16	2.02	11.93	>1000.00	>.999
VFCS					
Nonsense variant	-0.47	-2.53	1.62	0.5	0.671
Frameshift	-0.50	-2.55	1.57	0.5	0.686
Whole gene deletion	-2.07	-5.67	1.23	0.1	0.896
Splice	0.47	-2.03	2.89	1.8	0.645
EDACS					
Nonsense variant	3.94	-0.19	10.14	56.3	0.983
Frameshift	4.22	0.02	10.33	95.6	0.990
Whole gene deletion	5.48	0.54	11.97	123.2	0.992
Splice	4.99	0.51	11.32	157.7	0.994
